# Avatars versus the people: Photo-realism, spontaneous detection and trait inferences of digitised faces

**DOI:** 10.1177/03010066251379016

**Published:** 2025-09-29

**Authors:** Kyara C. Nasser Oesterreich, Matthew C. Fysh, Markus Bindemann

**Affiliations:** 1 2240 University of Kent, United Kingdom; 2 The Tavistock and Portman, NHS Foundation Trust, United Kingdom

**Keywords:** face perception, avatar, realism, detection, trait inferences, eye-tracking

## Abstract

Technologies aiming to imitate human faces are becoming increasingly realistic. This study investigates a facial imitation technology that is becoming widespread – digital characters of people for presentation in virtual reality. Avatar faces were created from high-resolution 3D scans of real people. Across a series of four experiments, the photo-realism of these avatar faces was compared with passport-style face photographs of the same persons. In Experiments 1 and 2, these stimuli could be distinguished with high accuracy when a direct comparison of avatars and photographs was possible. In contrast, discrimination accuracy decreased when avatars and photographs were encountered in isolation, while awareness that avatar faces had been encountered was also low. Experiments 3 and 4 showed that avatars and face photographs generate similar trait inferences of attractiveness, dominance and trustworthiness. In cases where differences between avatars and photographs emerge, analysis of viewing patterns indicates that these originate from the eye region of these stimuli, which receive more fixations in avatars than face photographs. These findings demonstrate that the visual realism of avatars can closely resemble that of face photographs, particularly in contexts in which realism is not explicitly evaluated. Differences between avatars and photographs become more apparent when participants are cognizant and able to make direct comparisons.

## Introduction

Technologies aiming to imitate human faces are becoming increasingly realistic. Such imitation can be achieved through physical transformation. Hyper-realistic silicon masks, for example, can be worn by a person over their head to transform their facial appearance. Psychological studies show that viewers frequently fail to detect the latest generation of these masks. This occurs across a range of settings, such as in comparison with photos of real faces in laboratory experiments (Sanders et al., 2019; Sanders & Jenkins, 2018), during interpersonal encounters in field studies (Robertson et al., 2020; Sanders et al., 2017), and in real-world criminal cases (Sanders & Jenkins, 2021). This demonstrates that it can be difficult to distinguish real from not-real faces. Another way to imitate facial appearance is through the digital presentation of faces (Groh et al., 2021; Hancock & Bailenson, 2021). Hyper-realistic visual imitations of real people can be created with software that are now widely available (e.g., FaceApp and FaceSwap). Videos of such ‘deepfakes’ are not only difficult to differentiate from authentic person footage, but viewers also overestimate their ability to do so (Köbis et al., 2021), further enhancing the potential threats that such manipulations can pose.

Here, we investigate another facial imitation technology that is becoming increasingly widespread and capable – digital representations of real people depicted in virtual reality (VR). With VR, viewers can be immersed in audio-visual environments that are increasingly complex, detailed and realistic. Over the last decade, VR has become a mainstream technology in the entertainment industry, including gaming, cinema, and tourism. In parallel with such developments, VR is also becoming increasingly useful for conducting psychological research, due to its capacity to simulate real-world environments (see, e.g., Pan & Hamilton, 2018). Yet, a key challenge encountered throughout all of these domains is the development of the digital people that populate VR settings. The gaming and entertainment sectors demonstrate that it is possible to create high-quality digital characters, called avatars, that are based on real people with increasing fidelity. As such technologies become increasingly available, it has become possible for psychologists with an interest in person perception to create avatars of real people with high fidelity, too.

We have recently developed such a method, based on the recording of 3D scans of real faces, which are then post-processed into animated digital avatars (Fysh et al., 2022). We have shown that these avatars are reliably identifiable by people who are familiar with their real-world counterparts. They can also be matched with high accuracy to a face photograph by viewers who are unfamiliar with these identities. Moreover, by performing a principal components analysis of avatar faces and photographs, we were able to map out the face-space of photos and avatars to show that people who look similar in photos also look similar as avatars. In addition, we have shown that identification of these avatars in a virtual reality airport setting correlates with established laboratory tests of person identification, indicating that their perception draws on the same cognitive processes as person identification from photographs (Bindemann et al., 2022).

What these experiments cannot reveal, however, is the extent to which viewers can distinguish whether they are presented with an avatar or a real person. This issue is important theoretically and practically. Psychological experiments that are successful at capturing key characteristics of the real-world carry greater ecological validity and allow for more meaningful theorising. The study of perspective-taking demonstrates, for example, that viewers are more likely to adopt the perspective of avatars under conditions of increased realism (Nielsen et al., 2015; O’Grady et al., 2020). Increasing such correspondence may also improve applications of avatar technologies, such as the user-experience of games or virtual meeting applications. In turn, imitation technologies can be abused for criminal purposes, and understanding the potential of VR for such uses will also help to define the scope of these problems.

In this study, we present four experiments to investigate this question. We do so by comparing images of avatars, which were created from 3D face scans of real people, with face photographs of the same person. This presentation differs from the implementation of avatars in gaming and VR, where these are presented in 3D as a complete person (i.e., with a body), are capable of movement, and seen in visual contexts that can provide other reality clues. Here, we present avatars as static 2D images to eliminate these additional information sources. This approach prioritises tight control in stimulus presentation over ecological validity, but provides an important comparison: Photographs are used for many important functions, such as identity verification in security settings. If high-quality photographs of real faces are taken as the benchmark for photo-realism (i.e., to provide a veridical and realistic representation of a person's face), then a comparison with these stimuli can reveal the extent to which it is possible to distinguish avatars from real people in image format. This will reveal whether the appearance of artificial person stimuli such as avatars successfully imitates real faces (Turing, 1950).

We assess viewers’ ability to distinguish avatars from faces in a number of ways. This includes realism ratings of avatar images and face photos as well as the forced-choice differentiation of these stimulus categories (Experiment 1), the spontaneous detection of avatar faces (Experiment 2), and the trait attributions that are made to avatars and face photos (Experiments 3 and 4). Human observers have consistently demonstrated a capacity for detecting small changes in face stimuli, such as judgements about subtle metric changes between facial features (Crookes & Hayward, 2012; Freire et al., 2000; Leder & Bruce, 2000), the presence of minute features (Fysh & Bindemann, 2022), or the differentiation of emotional expressions that morph slowly from one face stimulus to another (Calder et al., 1996; Etcoff & Magee, 1992; McKone et al., 2001), as well as social judgements that require sensitivity to a variety of differences between faces (Todorov et al., 2009; Willis & Todorov, 2006). Thus, one might also expect observers’ ability to differentiate images of avatars from photos of real faces to be high. On the other hand, the development of hyper-realistic masks (Robertson et al., 2020; Sanders et al., 2017, 2019; Sanders & Jenkins, 2021) and deepfakes (Groh et al., 2021; Hancock & Bailenson, 2021; Köbis et al., 2021) demonstrates the increasing power of artificial stimuli to imitate real faces. Here, we investigate whether avatars can also achieve convincing imitations.

## Experiment 1

This experiment examined viewers’ ability to distinguish images of avatar faces from photographs of real faces across a series of tasks. We begin with a ratings task, in which viewers were asked to judge the realism of faces on a 7-point scale to provide a fine-grained comparison of avatars and photographs. For comparison, we also included low-resolution images of avatars and photographs of faces to increase the difficulty with which these stimuli can be distinguished. This allowed us to determine whether realism ratings for avatars are more similar to these low-resolution images or the high-resolution face photographs. We did not inform participants of the inclusion of avatars in advance, which also allowed us to assess whether viewers spontaneously noticed the presence of these stimuli. For this purpose, we asked a series of graded avatar detection questions that were adapted from previous research on hyper-realistic masks (see Sanders et al., 2017). Finally, we included two forced-choice tasks, in which participants were asked directly to classify faces as photographs or avatar images. These judgements were first made to faces presented in isolation and later as avatar-photo pairings.

### Method

#### Participants

A total of 52 participants were recruited using Prolific (prolific.com) and were paid for participation. The sample size was based on similar research (see Sanders et al., 2017, 2019; Sanders & Jenkins, 2018) from which the methodologies were adapted. Two participants did not complete the study and were therefore excluded. The remaining sample consisted of 50 individuals (24 males, 24 females, 1 non-binary/third gender, 1 prefer not to say) with a mean age of 27.94 years (*SD* = 8.17). Participants self-reported their ethnicities as White (*N* = 33), Black (*N* = 11), Asian (*N* = 2) and Other (*N* = 4).

#### Stimuli

The avatars and photographs were taken from Fysh et al. (2022). Passport-style photographs of 40 models were collected with a high-quality digital camera (Fujifilm FinePix S2980, 14-megapixel), showing each person in a frontal pose, with neutral expression and under good lighting. These images were cropped of extraneous background and sized to 500 (w) × 500 (h) pixels at a resolution of 72 ppi. For each model, a corresponding avatar was created: In the same session that the face photograph was taken, every person was also scanned with a 3D scanner (Artec Eva). The raw scan was then processed by fusing its components into a single wireframe mesh that represented the subject's head geometry, followed by the application of texture. Following this step, each head scan was ‘wrapped’ to a standardised base geometry, which produced standard UV texture maps and a common 3D topology for each identity (for a detailed workflow, see Fysh et al., 2022).

The 3D scanner uses structured light to detect shape. Consequently, the resulting 3D faces are lit uniformly, and any shape-from-shadowing information is minimised. To match the face scans to the face photographs for lighting, we introduced lighting and shading with a 3D modelling/animation program (Blender). With this software, a base scene setup was created, with the head geometry positioned at the global origin point, corresponding to position zero across all three axes.

Next, a virtual camera was positioned 8 m away from the global origin on the *y*-axis, at a height of 1.8 m on the *z*-axis, and with a rotation of 90 degrees. The camera was in perspective mode with a focal length of 50 mm. A spotlight was then placed in the same position and rotation but offset by 1 m (closer to the global origin) on the *y*-axis, with a power of 1000 W. A pointlight was positioned six metres to the spotlight's left (−6 on the *x*-axis), at a height of 3.5 m on the *z*-axis, again with a power of 1000 W. This lighting setup roughly mimicked the light positioning used when taking the photographs of the individuals.

**Figure 1.**
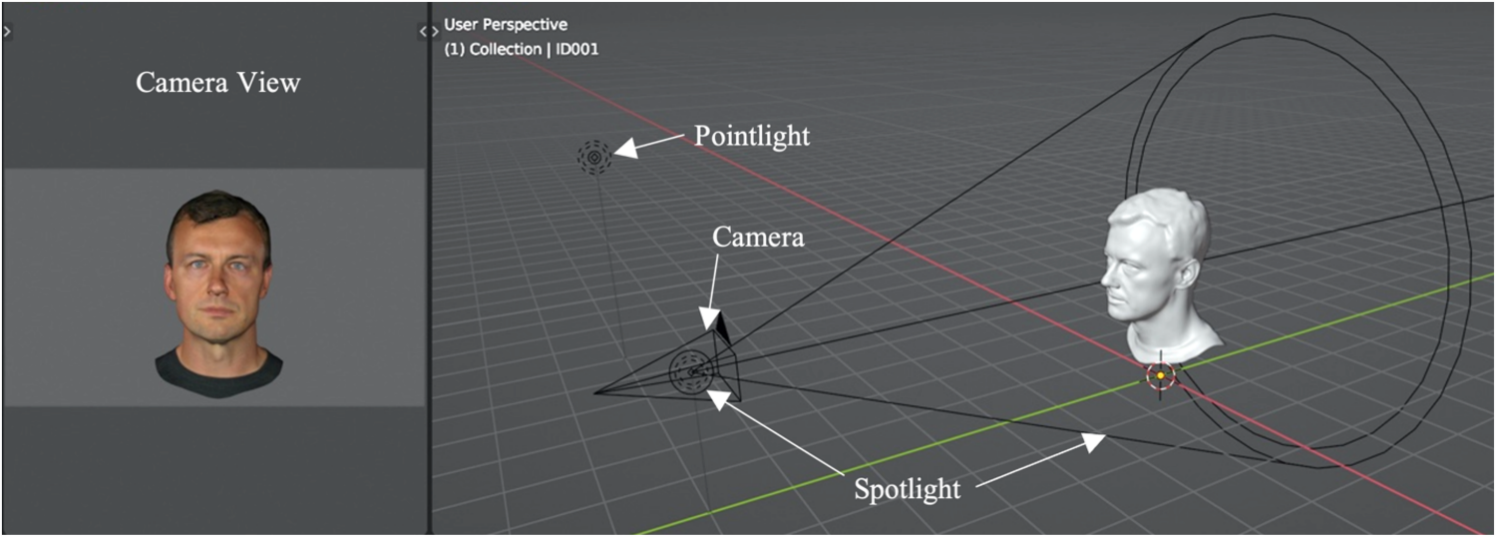
Base set-up for rendering the face images of avatars. Image of the user perspective (right) in object mode in solid view, displaying the camera, the spotlight and point light and an example avatar mesh. To the left is the camera view (in render view) displaying the avatar face in its end state. The brightness of this image has been increased slightly for illustration purposes.

Within this virtual set up, avatars were placed at world origin facing the camera directly. The heads were scaled to occupy most of the camera view, with sufficient space around the edge to allow for later editing. To ensure avatar positioning stayed consistent, all avatars were positioned based on the initially imported avatar. Smooth shading was used, and the texture image was applied using the default principled bsdf shader in Blender with the specular set to zero, since reflections and certain lighting information were already baked into the texture, and the material output was applied to the model's surface. With this setup, the high-quality images were rendered, using Blender's EEVEE renderer, that matched the face photographs in terms of lighting and shading. EEVEE was chosen to reflect how digital avatars are typically rendered in real-time applications. An illustration of this set up can be seen in Figure 1.

**Figure 2.**
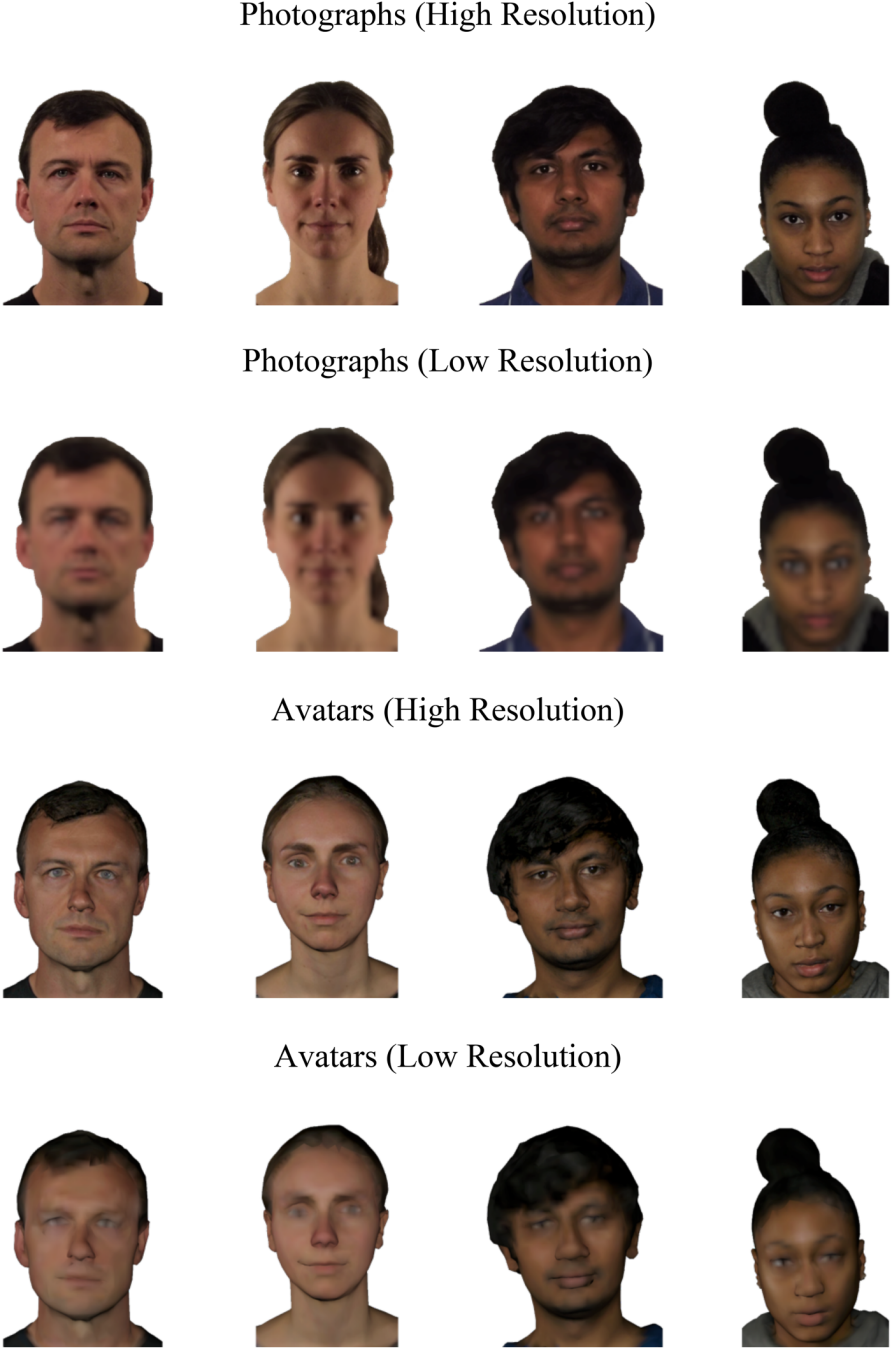
Example stimuli for photographs and avatars in the high- and low-resolution conditions.

To produce low-resolution versions of the face photos, the digital photographs were scaled down by 90% (i.e., to 50 × 50 pixels) and then rescaled to full height (500 × 500 pixels). For the avatars, a different method was used that allowed us to directly alter their structure and texture prior to rendering, rather than applying 2D degradation post-render to stay true to the nature of these stimuli. Thus, the detail of the three-dimensional shape was reduced by using the decimate modifier in Blender to collapse edge information systematically using a reduction ratio of 0.1. In parallel, texture quality was reduced by decreasing image resolution by 90%. With these alterations, the ‘low-resolution’ images of the avatars were rendered. Lastly, the grey background was cropped out of both the high-resolution renders and the low-resolution renders, and the renders were cropped to match the positioning of the photographs and were resized to 500 × 500 pixels at a resolution of 72 ppi. Example stimuli for all conditions can be viewed in Figure 2.

#### Procedure

##### Realism Ratings Task

All data collection was conducted online using Prolific (prolific.com). Consent and demographic information were recorded with an initial Qualtrics survey (qualtrics.com). Participants were then directed to the experiment, which was implemented on Pavlovia (pavlovia.org). As stimuli were displayed on participants’ personal computers, we included a screen calibration procedure to ensure the images would be presented at a standard size of 10 × 10 cm. For calibration, participants were asked to adjust the size of an onscreen rectangle to the size of a credit card. Participants were then presented with a face stimulus in the centre of the screen, with the caption ‘How realistic is this image?’, while a 7-point scale ranging from ‘*not at all*’ to ‘*completely*’ was presented below each face. Participants registered their response by mouse-clicking on the relevant scale point. The next stimulus then appeared in a randomised order. This procedure was repeated for all 40 identities and each of the experimental conditions (high-resolution avatar, high-resolution photo, low-resolution avatar, low-resolution photo), giving a total of 160 trials.

##### Spontaneous Avatar Detection

After rating all stimuli, four follow-up questions were asked. Question 1 and 2 probed for general impression of the faces (‘What did you think of the faces you saw?’ and ‘Did you notice anything unusual about the faces?’). These questions were deliberately open to capture spontaneous detection of avatars, without drawing attention to the distinction between photos and avatars. Question 3 then probed avatar detection directly (‘In this experiment some of the faces were digital photographs while others depicted avatars [digital human beings]. Did you notice that?’), and Question 4 asked participants to estimate how many (%) of the images were avatars (‘Of the faces that you saw, how many do you think were avatars?’). Participants responded to Question 1 and 2 by typing a response into text prompts onscreen, Question 3 required a yes or no response by pressing ‘y’ or ‘n’ on their keyboard, and Question 4 was answered by mouse clicking on a scale ranging from 0% to 100%.

##### Forced-Choice Classification (Individual Images)

After the detection questions, participants were again presented with the face images in a random order. This time, each trial consisted of a 0.5-second fixation cross, followed by a single face stimulus, which remained onscreen until a response was registered. Participants were asked to determine whether a stimulus was a photograph or an avatar by pressing one of two buttons on the keyboard. Participants completed 160 trials, comprising of 40 trials for each experimental condition (high-resolution avatar, high-resolution photo, low-resolution avatar, low-resolution photo).

##### Forced-Choice Classification (Paired)

This was followed by a final task, in which participants were presented simultaneously with the high-quality photo and avatar images of the same identity and were asked to indicate which of the two images was the digital photo in a Turing Test (Turing, 1950). The side on which the photo and avatar was presented was counterbalanced across trials, and trial order was randomised for each participant. The task consisted of 40 trials, comprising of 40 avatar-photo pairings in the high-resolution condition. All experiments reported here received approval from the School of Psychology Ethics Committee at the University of Kent (approval number 202216599642167870).

### Results

#### Realism Ratings for Photos and Avatars

**Figure 3.**
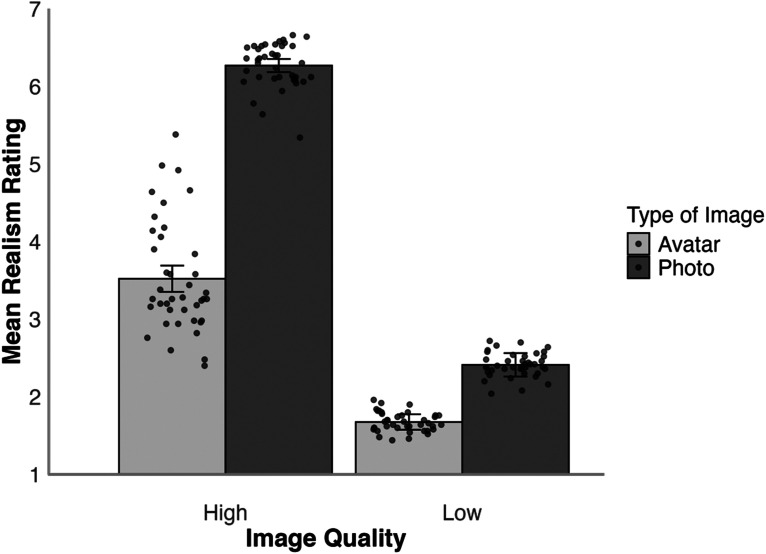
Average realism rating for high- and low-resolution avatars and photos in Experiment 1. Error bars denote standard error of the mean. The circular markers indicate the cross-subject mean rating for each stimulus identity.

The data for all experiments reported here can be accessed on OSF at https://osf.io/kgdta/?view_only=04525bf8ffb347e380207de9e83eb650. We first compared realism judgements for avatar images and digital photos. The mean realism ratings for these stimuli for the high- and low-resolution conditions are illustrated in Figure 3. A 2 (face type: photo, avatar) × 2 (resolution: high, low) within-subjects ANOVA of these data showed a main effect of resolution, *F*(1,49) = 645.87, *p* < .001, $\eta_{p}^{2}=0.93$ , due to higher realism ratings in the high- than the low-resolution conditions. A main effect of face type was also found, *F*(1,49) = 227.15, *p* < .001, $\eta_{p}^{2}=0.82$ , due to higher realism ratings for photos than avatars. This was qualified by an interaction between these factors, *F*(1,49) = 146.34, *p* < .001, $\eta_{p}^{2}=0.75$ . Tukey HSD Test showed that high-resolution images were rated as more realistic than low-resolution images, both in the photo, *t*(49) = 25.71, *p* < .001, *d* = 3.64, and the avatar condition, *t*(49) = 14.38, *p* < .001, *d* = 2.03. In addition, photos were also rated as more realistic than avatars, both under high, *t*(49) = 18.82, *p* < .001, *d* = 2.66, and low resolution, *t*(49) = 5.31, *p* < .001, *d* = 0.75.

#### Spontaneous Avatar Detection

To examine the overt detection of avatars, we then analysed the content of the typed responses to Question 1 (‘What do you think of the faces you saw?’) and Question 2 (‘Did you notice anything unusual about the faces?’). To avoid imposing interpretations, these responses were coded for the presence (1) or absence (0) of words relating to avatars. These words included terms such as ‘Avatar’, ‘3D Model’, ‘Render’, ‘Animation’ and ‘Computer-generated’ (including alternative phrasing such as CGI, generated by computer/AI, made by computer, etc.). This revealed that in response to Question 1, only 26.0% (*N* = 13/50) of participants noted the presence of avatars. One more individual displayed awareness of avatar presence in response to Question 2, suggesting that less than a third of individuals (28.0% or *N* = 14/50) spontaneously detected and reported the avatars. For Question 3, which directly asked whether participants had noticed the presence of avatars, 84.0% (*N* = 42/50) of participants responded that they had done so. Finally, in response to Question 4 (‘Of the faces that you saw, how many do you think were avatars?’), the mean response was 53.6% (*SD* = 19.1).

#### Forced-Choice Classification (Individual Images)

**Figure 4.**
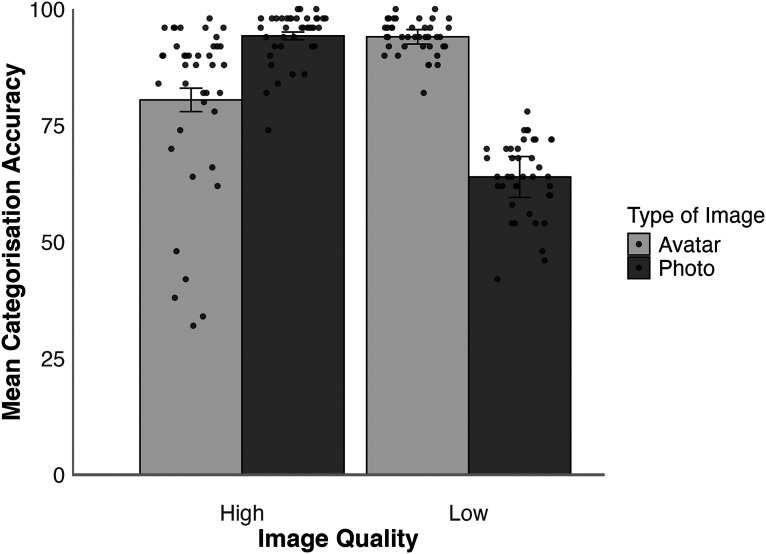
Categorisation accuracy for high- and low-resolution avatars and photos in Experiment 1. Error bars denote standard error of the mean. The circular markers indicate the cross-subject mean rating for each stimulus identity.

In this task, participants were shown one stimulus at a time and asked to categorise this as a photo or an avatar. The mean percentage of correct responses was analysed for the high- and low-resolution conditions and is illustrated in Figure 4. A 2 (face type: photo, avatar) × 2 (resolution: high, low) within-subjects ANOVA showed a main effect of resolution, *F*(1,49) = 17.24, *p* < .001, $\eta_{p}^{2}=0.26$ , due to higher accuracy in the high- than low-resolution conditions. A main effect of face type was also found, *F*(1,49) = 7.14, *p* = .010, $\eta_{p}^{2}=0.13$ , due to lower accuracy for photos than avatars. These effects were qualified by an interaction, *F*(1,49) = 75.60, *p* < .001, $\eta_{p}^{2}=0.61$ .

Tukey HSD Test showed that high-resolution photos were more accurately identified as digital photographs than low-resolution photos, *t*(49) = 7.36, *p* < .001, *d* = 1.04. In contrast, low-resolution avatars were more likely to be identified as avatars than high-resolution avatars, *t*(49) = 6.87, *p* < .001, *d* = 0.97. Moreover, high-resolution photos were more likely to be correctly identified than high-resolution avatars, *t*(49) = 5.07 *p* < .001 *d* = 0.72, whereas low-resolution avatars were more likely to be correctly identified than low-resolution photos, *t*(49) = 6.13, *p* < .001, *d* = 0.87.

These results show that low-resolution images were more likely to be classified as avatars compared to high-resolution images. This pattern may reflect the distinct approaches used to downsample avatars and photographs (reducing avatars’ 3D detail before rendering versus downsampling photographs after capture), which may have introduced visual differences between the two sets. In this case, the respective reduction methods reduced realism and increased avatar responses for avatars and photographs compared to the high-resolution counterparts. Accuracy for high-resolution face photographs approached ceiling, whereas high-resolution avatars were misclassified as photos of real faces on nearly one in five trials. We therefore also analysed whether these findings are consistent across stimuli. The extent to which avatars were mistaken as face photos varied considerably between the different stimulus identities. For high-resolution avatars, accuracy ranged from 32.0% to 98.0% across items. Put differently, one avatar was categorised as a photograph of a real face by 68.0% of participants, whereas another avatar was mistaken as a photograph by only one person (2.0%). However, all high-resolution avatar images were mistaken as photographs at least once. Moreover, in the realism ratings task, 66.0% of these images received the maximum realism rating of 7 from at least one participant, while the remaining 34.0% of avatars received a maximum rating of 6. As one would expect, higher realism ratings for the high-resolution avatars correlated with lower accuracy rates in the forced-choice task, *r*(38) = −.91, *p* < .001.

#### Forced-Choice Classification (Paired)

The final task involved the direct comparison by observers of side-by-side avatars and photos to determine which image was the avatar. Accuracy on this task was high (*M* = 94.1%, *SD* = 13.6). To establish whether performance was aided by viewing digital photographs and avatars simultaneously, these accuracy data were compared with the high-resolution condition of the sequential forced-choice task, which was collapsed across the photo and avatar conditions (*M* = 87.4%, *SD* = 9.2). A paired-samples t-test confirmed that accuracy was higher for forced-choice comparisons of photo-avatar pairings than when these stimuli were shown individually, *t*(49) = 2.95, *p* *=* .005, *d* = 0.42.

### Discussion

This experiment shows that images of avatars can be distinguished from photos of real faces with high accuracy under conditions that facilitate comparison, for example, when images of the same person are shown side-by-side in a forced-choice comparison. However, the accuracy of these decisions declines when such direct comparisons are made more challenging through the sequential presentation of photos and avatars, even though this occurred at a point of the experiment by which participants were aware of the inclusion of the avatars. Under these conditions, nearly every one in five avatars was misclassified as a photograph. Moreover, some avatars were consistently misclassified as photographs, and all avatars were perceived to be photos of real faces at least some of the time. Most strikingly, although avatars were rated to be less realistic than photographs, the spontaneous detection of these stimuli was low. Without direct prompts, only 28.0% of participants reported that an avatar-style stimulus might have been presented in the ratings task. Even when asked directly about the presence of avatars, 16.0% of participants still did not recognise that they had been presented with such stimuli. These findings suggest that the detection of avatars can be difficult depending on the task demands, especially if participants are unaware of the inclusion of such images or are unable to directly compare these with corresponding photographs of real faces.

## Experiment 2

Experiment 1 demonstrates that avatars are distinguished from photographs of real faces most accurately when these are compared directly. This raises the question of how the realism of avatars is perceived when direct comparisons are not possible, such as when participants only see face photographs *or* avatars. In addition, Experiment 1 also revealed differences between avatars in the likelihood that they passed for photographs of real people. Such variability in avatar realism might raise participants’ awareness that such stimuli are present in an experiment. In turn, it is possible that the realism of avatars is perceived to be similar to that of face photos when the most-realistic exemplars of these stimuli are shown first and direct comparisons with lower-realism avatars are not possible. In Experiment 2, we therefore assessed the realism of avatars by presenting these separately from photos (i.e., on a between-subject basis) and by manipulating the order in which stimuli were shown so that the identities reflecting avatars with the highest realism in Experiment 1 were always encountered first.

### Method

#### Participants

A total of 58 participants were recruited using Prolific and paid for participation. The sample size was again based on similar research (see Sanders & Jenkins, 2018; Sanders et al., 2017, 2019) from which the methodologies were adapted. Eight participants did not complete the study and were therefore excluded. The remaining sample consisted of 50 individuals (18 male, 32 female), with a mean age of 38.54 years (*SD* = 11.44). Self-reported ethnicities were White (*N* = 39), Black (*N* = 2), Asian (*N* = 7) and Other (*N* = 2).

#### Stimuli and Procedure

**Figure 5.**
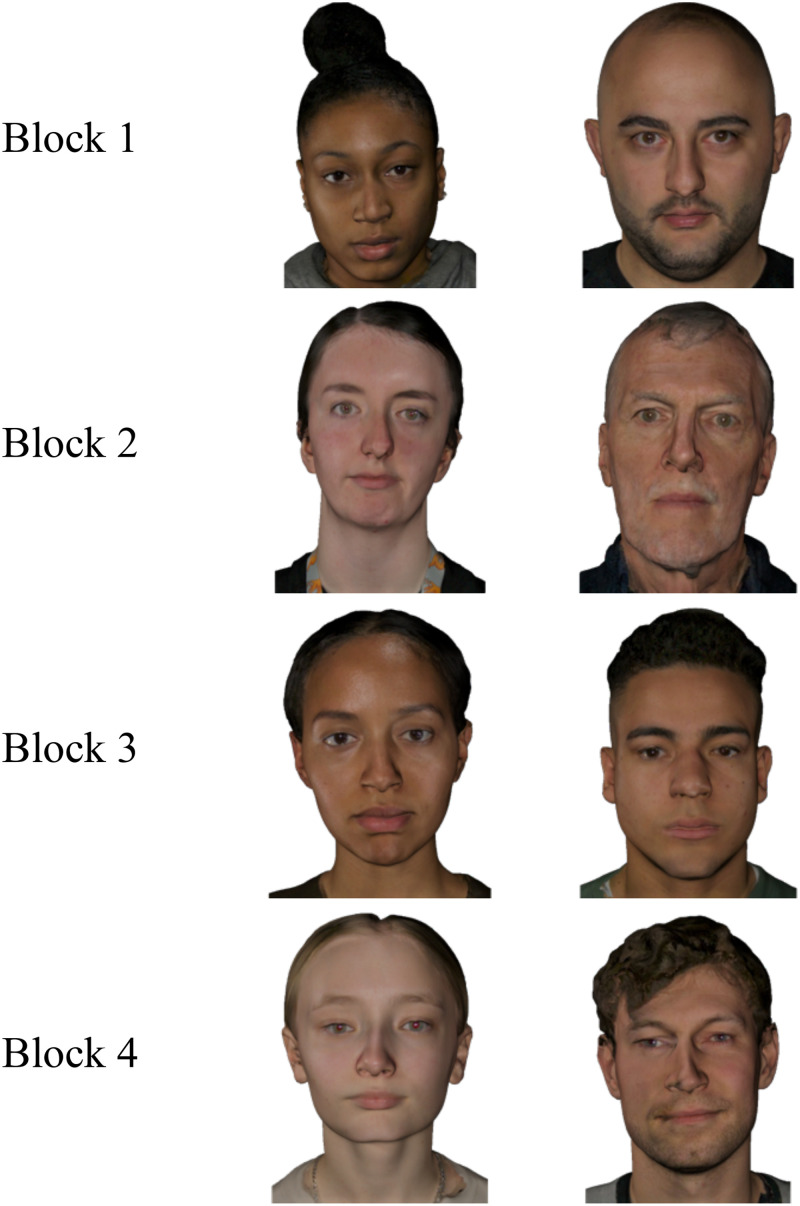
Examples of avatars from each block of Experiment 2.

The stimuli consisted of the 80 high-resolution images (i.e., 40 avatars, 40 face photographs) from Experiment 1. The avatar images were rank-ordered using two measures from Experiment 1: their average realism ratings and their classification accuracy. For realism ratings, avatars with higher ratings were given lower ranks (i.e., rank 1 = most realistic avatar in the set of 40, to rank 40 = least realistic avatar in the set). For classification accuracy, avatars that were more often mistaken for real photographs were also given lower ranks (i.e., rank 1 = most often misclassified to rank 40 = least often misclassified). The two ranks were then averaged to create a composite rank for each avatar. Based on this composite rank, avatars were divided into four blocks of decreasing realism. Each block consisted of 10 trials. Block 1 contained the ten avatars with the lowest (i.e., most realistic) composite ranks, and Block 4 contained the ten avatars with the highest (least realistic) ranks. The order of blocks was fixed across participants, progressing from the most to the least realistic avatars. Within each block, however, the order of stimulus presentation was randomised. We included a second condition consisting of high-quality face photographs, which were divided into blocks of 10 trials according to the block assignments of their corresponding avatar identities. The order of blocks and randomisation of stimuli within blocks followed the same structure as in the avatar condition. Example avatars for each block are illustrated in Figure 5.

In the experiment, participants were allocated to the avatar or photo condition on a between-subjects basis. They were then shown one face at a time, with the caption ‘How real is this image/person?’ and were asked to rate the realism on a 7-point scale. After each block, participants were asked two follow-up questions to probe the spontaneous detection of avatars (‘What did you think of the faces you saw?’ and ‘Did you notice anything unusual about the faces?’). After Block 4, participants were given two further questions to probe avatar detection directly (‘This experiment possibly contained faces that depicted avatars [digital human beings]. Did you notice any avatars?’ and ‘Of the faces that you saw in Block #, how many do you think were avatars?’), the latter of which was repeated once for each block.

### Results

#### Realism Ratings for Photos and Avatars

**Figure 6.**
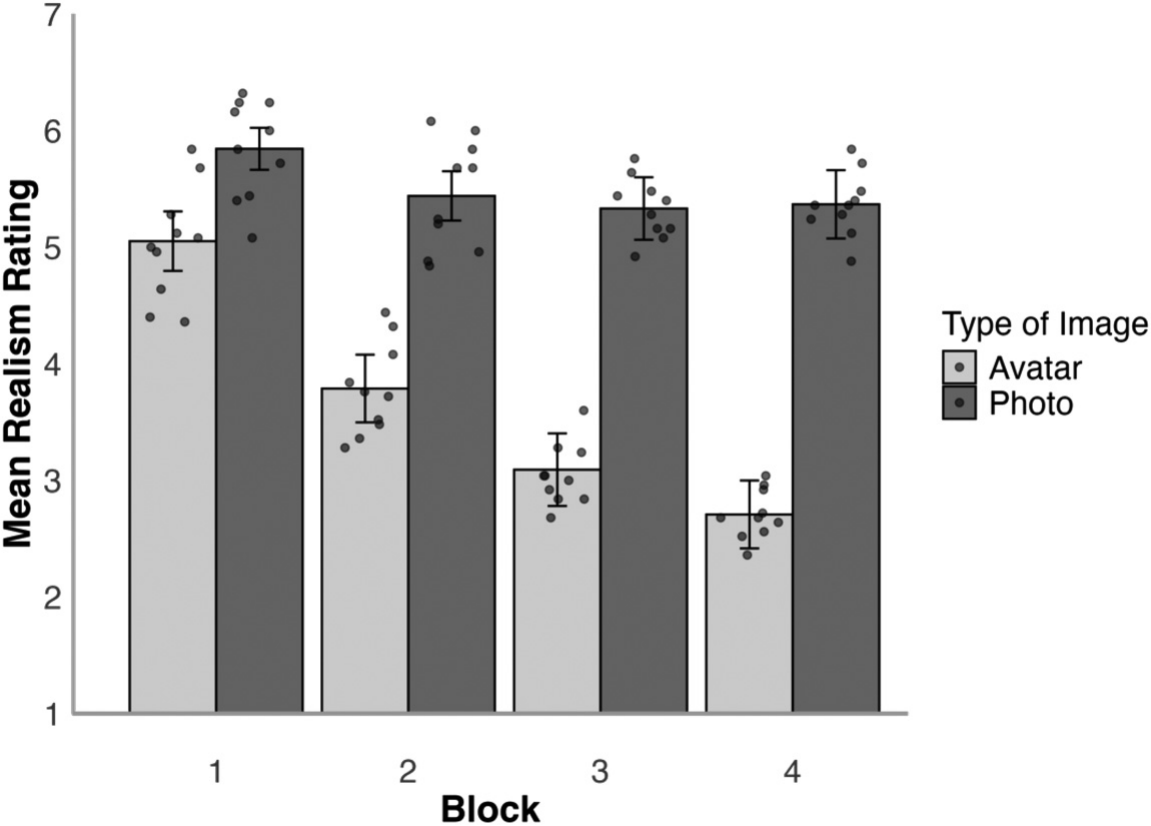
Average realism ratings for avatars and photos by presentation block in Experiment 2. Error bars denote standard error of the mean. The circular markers indicate the cross-subject mean rating for each stimulus identity.

We first examined the mean realism ratings for each block to compare realism judgements for avatar images and digital photos, which are illustrated in Figure 6. A 2 (face type: photo, avatar)×4 (block: 1, 2, 3, 4) mixed-factor ANOVA of these data revealed a main effect of block, *F*(3,144) = 37.44, *p* < .001, $\eta_{p}^{2}=0.44$ , due to lower realism ratings with each consecutive block. A main effect of face type was also found, *F*(1,48) = 30.70, *p* < .001, $\eta_{p}^{2}=0.39$ , due to higher realism ratings for photos than avatars. This was qualified by an interaction of block and face type, *F*(3,144) = 15.55, *p* < .001, $\eta_{p}^{2}=0.25$ . Tukey HSD Test showed that avatars were rated as more realistic in Block 1 than in Block 2, *t*(48) = 6.22, *p* < .001, *d* = 0.90, and in Block 2 than in Block 3, *t*(48) = 4.52, *p* < .001, *d* = 0.83, with comparable realism ratings for Block 3 and Block 4, *t*(48) = 2.58, *p* = .188, *d* = 0.41. In contrast, realism ratings were consistently high across all blocks for photos, all *ts*(48) ≤ 2.00, all *ps* ≥ .494, all *ds* ≤ 1.23*.* Correspondingly, Tukey HSD Test revealed no differences between photos and avatars in Block 1, *t*(48) = 2.54, *p* *=* .205, *d* = 0.72, whereas realism ratings were lower for avatars than photos in Block 2, *t*(48) = 4.60, *p* < .001, *d* = 1.30, Block 3, *t*(48) = 5.45, *p* < .001, *d* = 1.54, and Block 4, *t*(48) = 6.44, *p* < .001, *d* = 1.82.

#### Self-Reported Avatar Detection

As in Experiment 1, the detection of avatars was also examined with a series of questions. Question 1 (‘What do you think of the faces you saw?’) and Question 2 (‘Did you notice anything unusual about the faces?’) probed spontaneous detection of avatars. As in Experiment 1, participants’ responses to these questions were examined for the presence of words such as ‘Avatar’, ‘3D Model’, ‘Render’, ‘Animation’, and ‘Computer-generated’ (including alternative phrasing such as CGI, generated by computer/AI, made by computer etc.), in which case they were deemed to have detected the avatars. To analyse these responses, we combined the data for both of these questions and calculated the percentage of participants who had detected avatars in each block. For Block 1 to 4 of the avatars condition, this corresponded to 20.0%, 0.0%, 16.0% and 40.0% of participants. A repeated-measures non-parametric ANOVA (Friedman) revealed an effect of block, *χ*^2^(3) = 14.16, *p* = .003. Durbin Conover tests for pairwise comparisons revealed differences between all blocks, all *ps* ≤ .046, except between Block 1 and 3, *p* = .686, and Block 2 and 3, *p* = .108. These percentages reflect the number of participants who spontaneously reported avatars for each block, but do not take into account the consistency of participants’ responses across blocks. Therefore, we also calculated the cumulative percentage of these responses across blocks. For Block 1 to 4, these corresponded to 20.0%, 20.0%, 32.0% and 52.0% of participants. A repeated-measures non-parametric ANOVA (Friedman) revealed an effect of block, *χ*^2^(3) = 19.00, *p* < .001. Durbin Conover tests for pairwise comparisons revealed differences between Blocks 1, 2 and 3 in comparison with Block 4, all *ps* ≤ .009, but not between Blocks 1, 2 and 3, all *ps* ≥ .113.

For comparison, we asked participants the same question in the photo conditions. Here, 0.0%, 4.0%, 8.0% and 8.0% spontaneously used words that suggested the detection of avatars, despite the fact that they had seen none. A repeated-measures non-parametric ANOVA (Friedman) of these data did not show an effect of block, *χ*^2^(3) = 2.54, *p* = .468. Again, we also calculated the cumulative percentage of these responses, which were 0.0%, 4.0%, 12.0% and 16.0% across the four blocks. A repeated-measures non-parametric ANOVA (Friedman) of these data showed an effect of block, *χ*^2^(3) = 8.57, *p* = .036. Durbin Conover pairwise comparisons showed that the cumulative percentage of avatar responses was higher in Block 3 and 4 than in Block 1, both *ps* ≤ .045, and in Block 4 than in Block 2, *p* = .045. The remaining differences were not significant, all *ps* ≥ .177.

Next, we compared the percentage of ‘avatar’ responses for photos and avatars directly for each block using Kruskal-Wallis tests. This showed that more avatar reports were made in the avatar condition than the photo condition in Block 1, *χ*^2^(1) = 5.44, *p* = .020, and Block 4, *χ*^2^(1) = 6.88, *p* = .009, but not in Block 2, *χ*^2^(1) = 1.00, *p* = .317, and Block 3, *χ*^2^(1) = 0.74, *p* = .389. Similarly, a comparison of the cumulative percentages showed more avatar reports were made in the avatar than the photo condition in Block 1, *χ*^2^(1) = 5.44, *p* = .020, and Block 4, *χ*^2^(1) = 7.08, *p* = .008, but not in Block 2, *χ*^2^(1) = 2.97, *p* = .085, and Block 3, *χ*^2^(1) = 2.86, *p* = .091.

Finally, we examined responses to Question 3 (‘This experiment possibly contained faces that depicted avatars. Did you notice any avatars?’) and Question 4 (‘Of the faces that you saw in Block #, how many do you think were avatars?’). The data showed that 76.0% (*N* = 19/25) of participants in the avatar condition and 48.0% (*N* = 12/25) of participants in the photo condition responded that they had detected avatars, while the mean estimate of the proportion of seen avatars was 46.8% (*SD* = 9.1) in the avatar condition and 23.7% (*SD* = 5.8) in the photo condition.

**Figure 7.**
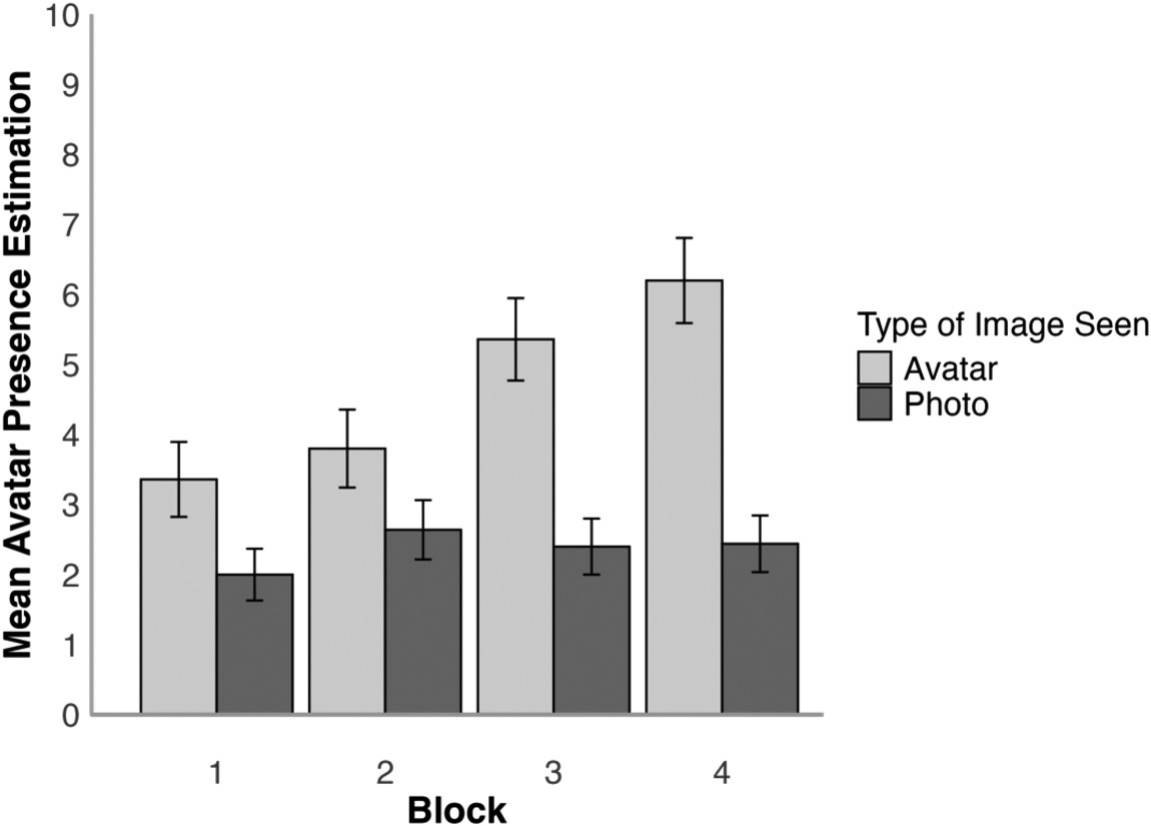
Mean estimated avatar presence per block for avatar and photo conditions in Experiment 2. Error bars denote standard error of the mean.

To analyse whether the estimation of avatar presence increased as the realism decreased, the mean estimated avatar presence was analysed using a 2 (face type: photo, avatar)×4 (block: 1, 2, 3, 4) mixed-factor ANOVA, which is illustrated in Figure 7. This revealed a main effect of block, *F*(3,144) = 7.86, *p* < .001, $\eta_{p}^{2}=0.14$ , due to later blocks being estimated to have more avatars than earlier blocks and a main effect of face type, *F*(1,48) = 18.47, *p* < .001, $\eta_{p}^{2}=0.28$ , due to more avatars being estimated to be present for the avatar condition. This was qualified by an interaction of block and face type, *F*(3,144) = 5.96, *p* < .001, $\eta_{p}^{2}=0.11$ .

Tukey HSD Test showed that there was no difference between avatar presence estimations for all blocks in the photo condition, all *ts*(48) ≤ 1.72, all *ps* ≥ .674, all *ds* ≤ 0.49, with the average amount of estimated avatars being 2.37 per block. In the avatar condition, there was a significant increase in estimated avatar presence between Block 1 and Block 3, *t*(48) = 4.16, *p* = .003, *d* = 0.81, Block 1 and 4, *t*(48) = 4.76, *p* < .001, *d* = 0.85, as well as between Block 2 and 4, *t*(48) = 3.53, *p* = .020, *d* = 0.62. None of the other comparisons between blocks within the avatar conditions were significant, all *ts*(48) ≤ 3.08, all *ps* ≥ .063, all *ds* ≤ 0.62. The comparisons for block within face type showed that there was no difference in the amount of estimated avatars between the face type conditions for Block 1 and 2, all *ts*(48) ≤ 2.09, all *ps* ≥ .435, all *ds* ≤ 0.59, but there was a difference within Block 3, *t*(48) = 4.16, *p* = .003, *d* = 1.18, and Block 4, *t*(48) = 5.15, *p* < .001, *d* = 1.46, due to avatar presence estimations being higher in the avatar condition.

### Discussion

Experiment 2 shows that the most realistic avatars from Experiment 1 are rated to be of a realism that is similar to face photographs. The spontaneous detection of these avatars is also low, with 80.0% of participants not noting anything unusual about these faces in Block 1. This pattern continued even in subsequent blocks, which employed avatars of decreasing realism, with nearly half of all participants (48.0%) still failing to spontaneously report the detection of avatars by the end of the experiment. Indeed, in the photo condition 16.0% of participants also made similar reports, suggesting that the gap between avatars is narrower still. Overall, these findings converge with Experiment 1 to demonstrate that avatars can appear to be highly realistic in 2D images and difficult to detect. These effects are particularly marked for the best avatars (presented in Block 1) produced with current methods (Fysh et al., 2022).

## Experiment 3

The preceding experiments demonstrate that some avatars can match the realism of photographs and that these stimuli can generally be difficult to detect spontaneously. Therefore, avatars should also perform similarly to photographs of real faces in other tasks. In this experiment, we examine this by investigating the impressions that observers form from faces. The formation of impression judgements from faces occurs rapidly and with a high level of agreement across individuals (Todorov et al., 2009; Willis & Todorov, 2006). While the attribution of many different traits has been studied, these can be captured by a smaller number of underlying dimensions, reflecting ‘attractiveness’, ‘dominance’ and ‘trustworthiness’ (Oosterhof & Todorov, 2008; Sutherland et al., 2013). However, these ratings are highly dependent on the specific photographic image of any individual. Moment-to-moment changes in facial expressions, for example, can introduce variability into the appearance of a person's face and this can affect social judgements (Jenkins et al., 2011; Lavan et al., 2021; Mileva et al., 2019; Todorov & Porter, 2014; Young, 2018). Here, we examine whether differences between a photograph of a person and an image of their avatar also affects these impression judgements. For this purpose, participants made attractiveness, dominance and trustworthiness judgements to the set of avatars and face photographs. We then examined the correspondence of these ratings.

### Method

#### Participants

A total of 67 participants were recruited using Prolific and paid for participation. Seven participants did not complete the study and were therefore excluded. The remaining sample consisted of 60 individuals (27 male, 33 female) with a mean age of 35.93 years (*SD* = 10.41). The participants self-reported their ethnicities to be White (*N* = 52), Black (*N* = 2), Asian (*N* = 3) and Other (*N* = 3). The sample size was increased from the previous experiments to allow for equal counterbalancing, and it generally corresponds to samples used in other studies utilising similar methodologies (see Sanders et al., 2017).

#### Stimuli and Procedure

The stimuli consisted of the 40 high-resolution avatar images and 40 high-resolution face photos from Experiments 1 and 2. Participants were randomly assigned to an avatar or photo condition. In these conditions, the respective stimulus sets were repeated across three blocks of 40 trials each (120 trials total). In these blocks, participants were asked to rate the attractiveness, dominance or trustworthiness of these stimuli on 7-point scales. For this purpose, a face stimulus was presented at the centre of the screen at a standardised size of 10 × 10 cm with the caption ‘How attractive/dominant/trustworthy is this person?’ and a 7-point scale ranging from ‘*not at all*’ to ‘*completely/extremely*’. Trial order was randomised within blocks and the order of blocks was counterbalanced across participants.

On completion of these blocks, three follow-up questions were asked, corresponding to Question 1 (‘What do you think of the faces you saw?’) and Question 2 (‘Did you notice anything unusual about the faces?’) from the preceding experiments to probe spontaneous detection of avatars. In addition, participants were also asked ‘In this experiment half of the participants saw digital photographs of real people and the other half of participants saw images of avatars (digital human beings). Which group do you think you were in?’.

### Results

**Figure 8.**
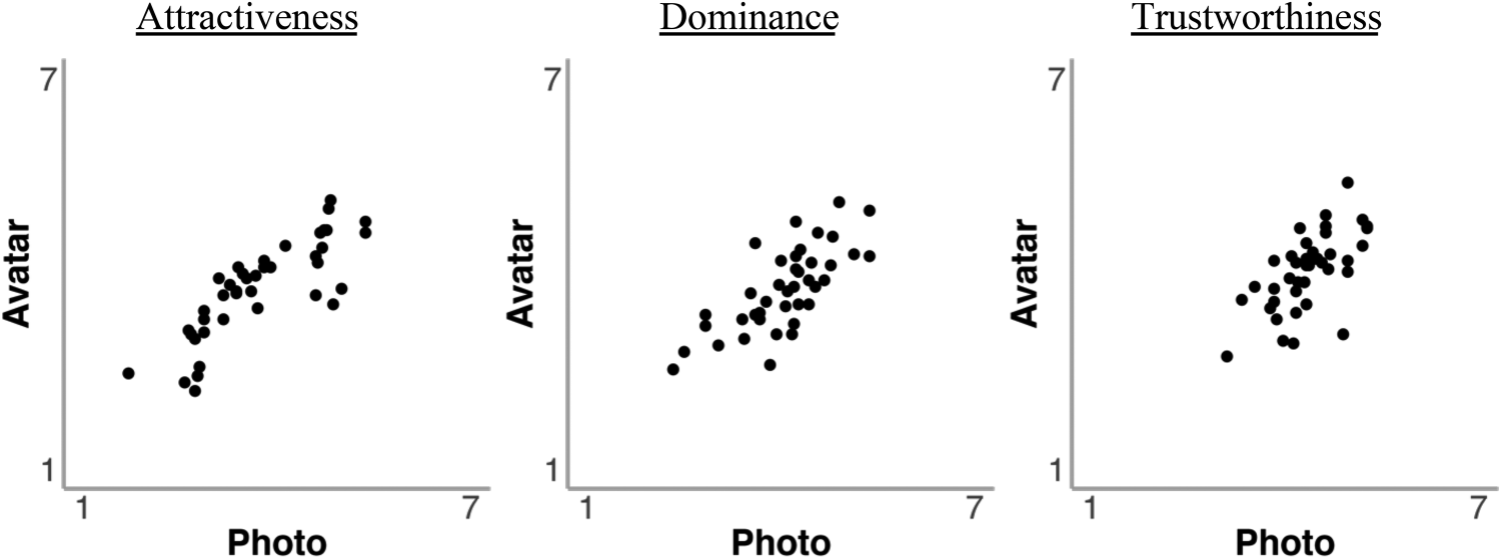
Correlations of attractiveness, dominance and trustworthiness ratings for avatars and photos in Experiment 3.

The mean attractiveness, dominance and trustworthiness ratings for avatar images and digital photos were compared with a series of independent-samples *t*-tests. These showed that these ratings were comparable for attractiveness (*M*_Photo_ = 3.71, *SD* = 0.77 vs. *M*_Avatar_ = 3.76, *SD* = 0.96), *t*(58) = 0.22, *p* = .825, *d* = 0.06, dominance (*M*_Photo_ = 4.04, *SD* = 0.46 vs. *M*_Avatar_ = 3.76, *SD* = 0.76), *t*(58) = 1.74, *p* = .088, *d* = 0.45, and trustworthiness (*M*_Photo_ = 4.36, *SD* = 0.55 vs. *M*_Avatar_ = 4.04, *SD* = 0.95), *t*(58) = 1.56, *p* = .125, *d* = 0.40. To further assess correspondence between these judgements for photos and avatars, the correlation of these ratings was also calculated. This revealed reliable correlations between ratings of avatars and photos for attractiveness, *r*(38) = .82, *p* < .001, dominance, *r*(38) = .73, *p* < .001, and trustworthiness, *r*(38) = .63, *p* < .001. These correlations are illustrated in Figure 8.

#### Self-Reported Avatar Detection

We again examined overt detection of avatars with a series of questions. Question 1 (‘What do you think of the faces you saw?’) and Question 2 (‘Did you notice anything unusual about the faces?’) probed spontaneous detection of avatars. As in Experiment 2, we combined responses for both questions. This showed that 36.7% of participants (*N* = 11/30) spontaneously reported such detection in the avatar condition, whereas 6.7% of participants (*N* = 2/30) made similar reports in the photo condition. As for Question 3 (‘In this experiment half of the participants saw digital photographs of real people and the other half of participants saw images of avatars [digital human beings]. Which group do you think you were in?’), responses showed that 76.7% (*N* = 23/30) of participants in the avatar condition and 13.3% (*N* = 4/30) of participants in the photo condition responded that they had been in the avatar condition.

### Discussion

This experiment demonstrates that facial judgements of attractiveness, dominance and trustworthiness correspond strongly for avatars and photographs. Therefore, avatars resemble photographs of the same identities in terms of the trait attributions that are made to faces. Once again, these observations were made in a context in which the spontaneous detection of avatars was low.

## Experiment 4

The low detection rate of avatars and the comparable error rates for correct identification of images from the previous experiments raise the question of whether photos and avatars are also scanned similarly during categorisation and trait attribution judgements. Eye-tracking shows that face perception often follows a T-shaped pattern, whereby the eye and mouth regions receive most attention during identity, emotion, and personality judgements (Diego-Mas et al., 2020; Gobel et al., 2015; Klein et al., 2009; Leopold & Rhodes, 2010; Schilbach, 2015). However, during the viewing of avatars such patterns might be disrupted by other image aspects, such as artifacts that might arise during their construction. This experiment therefore examined visual scanning patterns by recording observers’ eye movements to explore whether viewing differences exist between avatars and face photographs.

### Method

#### Participants

A total of 30 participants were recruited from a student sample who volunteered to participate in exchange for course credit. This sample was based on previous work with similar paradigms (see Sanders & Jenkins, 2018). The sample included 3 males, 26 females, and 1 non-binary/third gender participant. The participants had a mean age of 19.57 years (*SD* = 1.22) and self-reported their ethnicities as White (*N* = 19), Black (*N* = 4), Asian (*N* = 5) and Other (*N* = 2).

#### Stimuli and Procedure

The stimuli consisted of the 40 high-resolution avatar images and 40 high-resolution face photos from Experiments 1, 2 and 3, which were presented at a size of 20 × 20 cm and a screen resolution of 72 ppi. In the experiment, the stimuli were presented using SR-Research Experiment-Builder software (Version 2.2.61) on a 21-inch colour monitor connected to an SR-Research EyeLink 1000 desk-mounted eye tracking system, operating at a sampling rate of 500 Hz. Participants viewed the stimuli binocularly, but only the left eye was tracked. To calibrate the eye tracker, participants fixated on a series of nine fixation dots displayed on the screen using the standard EyeLink procedure. Thus, calibration accuracy was verified by fixating on a second sequence of fixation dots. If poor measurement accuracy was detected (with an average spatial precision of less than 1 degree of visual angle), the calibration process was repeated. This calibration procedure was conducted at the beginning of the experiment and prior to each new task.

The experiment consisted of four of the previous tasks: one categorisation task, in which faces were identified either as photographs or avatars via two keyboard buttons, and three trait attribution tasks, in which participants rated the attractiveness, dominance, or trustworthiness of the stimuli on 7-point scales ranging from 1 (*not at all*) to 7 (*completely/extremely*). Each task consisted of 80 trials in which participants viewed photographs and avatars of the 40 identities presented individually in a randomised order. The categorisation task always preceded the three trait attribution tasks, which were counterbalanced in order across participants. In each task, a trial began with the presentation of a central dot. Once participants fixated on the dot, the experimenter initiated the trial by pressing a button. A face stimulus was then displayed until a response was registered via participants’ button presses.

### Results

#### Categorisation Accuracy

First, participants’ ability to correctly identify and categorise face photographs and images of avatars was investigated. A paired-samples *t*-test comparing the mean percentage of correct judgements revealed no significant difference in accuracy between photos (*M* = 87.42, *SD* = 10.01) and avatars (*M* = 92.33, *SD* = 8.28), *t*(29) = 1.95, *p* = .061, *d* = 0.36.

#### Trait Judgements

Paired-samples *t*-tests were conducted to compare the mean ratings of attractiveness, dominance, and trustworthiness between avatar images and digital photos. The ratings for dominance were found to be comparable between photos and avatars (*M*_Photo_ = 3.91, *SD* = 1.35 vs. *M*_Avatar_ = 3.65, *SD* = 1.22), *t*(29) = 0.70, *p* = .493, *d* = 0.13, and also correlated strongly on a by-item basis, *r*(38) = .81, *p* < .001. The corresponding by-item analyses also showed a strong correlation for attractiveness, *r*(38) = .82, *p* < .001, and trustworthiness, *r*(38) = .58, *p* < .001, however, photos received higher ratings than avatars for attractiveness (*M*_Photo_ = 4.35, *SD* = 0.67 vs. *M*_Avatar_ = 2.76, *SD* = 0.63), *t*(29) = 9.90, *p* < .001, *d* = 1.81, and trustworthiness (*M*_Photo_ = 4.42, *SD* = 1.07 vs. *M*_Avatar_ = 3.07, *SD* = 1.08), *t*(29) = 4.11, *p* < .001, *d* = 0.75.

#### Eye Movements

**Figure 9.**
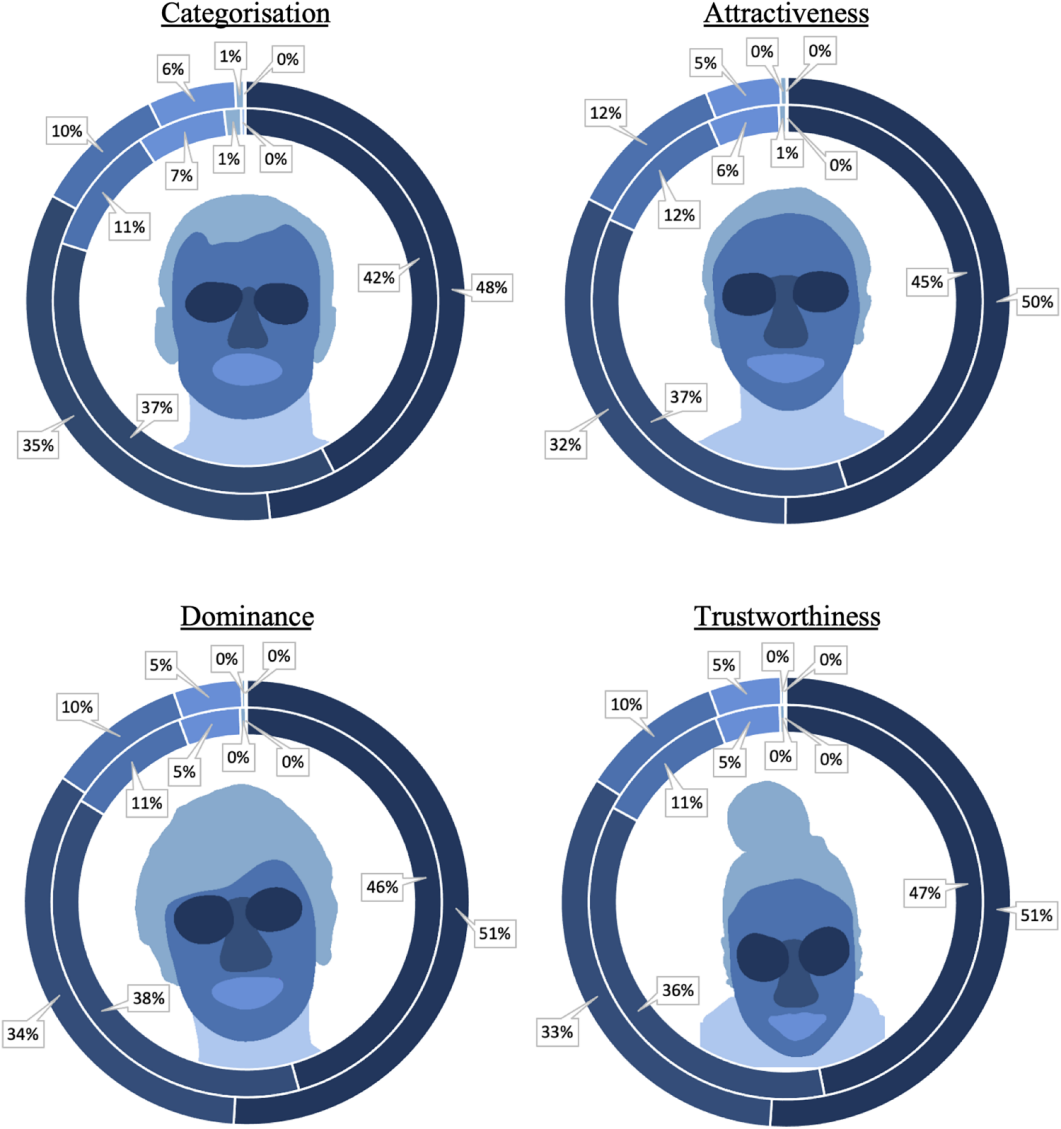
Percentage of fixations per face region by image type. The centre of each diagram shows an example of a region of interest map, with darker colours indicating more fixations on that region. The inside circle depicts the percentage of fixations on each feature for photographs, and the outside circle depicts the percentages for avatars.

Eye movements were analysed from the onset of the face stimuli. Fixations that fell outside the screen were excluded from the analysis. In addition, fixations lasting less than 80 milliseconds were integrated with either the preceding or subsequent fixation, if these fell within a radius of 0.5 degrees of visual angle, as such fixations might reflect false saccadic planning (see Rayner & Pollatsek, 1994). To investigate whether specific facial features were utilised differently in categorisation judgements between avatars and photographs, a region of interest (ROI) analysis was conducted. The analysis focused on examining the number of fixations directed towards the eyes, nose and mouth regions. These ROIs were determined individually for each face stimulus and were consistent with those defined and employed in previous research (e.g., Althoff & Cohen, 1999; Bindemann et al., 2009; Henderson et al., 2005). The remaining visible area of the face was defined as either face, hair (including ears if visible), or neck (including shoulders). An illustration of ROIs is provided in Figure 9.

##### Categorisation

The mean percentages of fixations on the ROIs of photos and avatars during the categorisation task are illustrated in Figure 9. A 2 (face type: photo, avatar) × 6 (feature: eyes, nose, face, mouth, hair, neck) within-subjects ANOVA of these data revealed a main effect of face type, *F*(1,29) = 5.77, *p* = .023, $\eta_{p}^{2}=0.17$ , a main effect of feature, *F*(5,145) = 85.95, *p* < .001, $\eta_{p}^{2}=0.75$ , and an interaction between these factors, *F*(5,145) = 11.37, *p* < .001, $\eta_{p}^{2}=0.28$ . The key differences are summarised here. As can be seen in Figure 9, the eyes and nose received by far the most fixations during the categorisation task. When fixations to avatar faces and photographs were compared directly with Tukey HSD test, only a single difference emerged, whereby the eye regions were fixated more frequently in avatars than in photographs, *t*(29) = 4.83, *p* = .002, *d* = 0.88. A summary of all comparisons is provided in Appendix A (Table A.1).

##### Ratings Tasks

An analogous analysis was performed for the ratings tasks. This revealed interactions of face type and feature for attractiveness ratings, *F*(5,145) = 11.32, *p* < .001, $\eta_{p}^{2}=0.28$ , trustworthiness, *F*(5,145) = 7.33, *p* < .001, $\eta_{p}^{2}=0.20$ , and dominance, *F*(5,145) = 17.44, *p* < .001, $\eta_{p}^{2}=0.38$ . All three tasks followed a similar pattern, whereby the eyes were the most fixated feature, followed by the nose, non-specific face regions, mouth and hair. However, only a few direct differences were found in fixation patterns between avatar faces and photographs, whereby the eye regions of avatars were looked at more often than those of photographs during attractiveness, *t*(29) = 4.06, *p* = .014, *d* = 0.74, trustworthiness, *t*(29) = 3.87, *p* = .023, *d* = 0.71, and dominance ratings, *t*(29) = 4.96, *p* = .001, *d* = 0.91. In addition, the nose region received fewer fixations in avatars than photos, but this effect was only reliable for dominance ratings, *t*(29) = 4.20, *p* = .010, *d* = 0.77. A summary of all comparisons is provided in Appendix A (i.e., Table A.2. for attractiveness, Table A.3. for trustworthiness and Table A.4. for dominance judgements).

### Discussion

This experiment demonstrates that both the categorisation of avatar faces and face photographs, and the attribution of traits based on their facial features, involve the majority of attention being directed towards the eye and nose regions. Moreover, only one consistent difference in viewing behaviour between these image types was observed, whereby the eye regions of avatars were inspected more than those of face photographs. In contrast, the nose regions of avatars received consistently less attention than those of photographs during dominance ratings. A similar numerical pattern was observed with the other tasks, but it was not reliable. However, this experiment only partially replicated the trait judgements of Experiment 3 in which photos and avatars received comparable dominance, trust and attractiveness ratings. In Experiment 4, only dominance ratings were comparable for these stimuli, whereas attractiveness and trustworthiness judgements for avatars were lower than for photographs. In Experiment 3, participants were only shown avatars *or* face photographs, whereas these stimuli were intermixed in Experiment 4. This suggests that the direct contrast between these stimuli can induce changes in attributions of attractiveness and trustworthiness.

## General Discussion

Avatars that represent individuals who exist in the real world have been employed increasingly in scientific research. This study assessed the realism of such avatars to determine the extent to which observers can detect these stimuli, both spontaneously and when explicitly required to do so. This was examined across four experiments by comparing images of avatar faces with real photographs of the corresponding identities.

In Experiment 1, participants distinguished avatars from real faces with a success rate of around 94.1% when these were presented side-by-side. However, discrimination accuracy decreased to around 87.4% when participants were exposed to these stimuli individually, so that a direct comparison of avatars and photos was not possible. Moreover, certain avatars were consistently misclassified as face photographs, and each avatar image was mistaken at least once as a real photograph during the experiment. In addition, only about a quarter of participants (28.0%) spontaneously noticed the presence of avatars in this task, and one in six observers (16.0%) still failed to recognise that avatars had been shown when asked directly about their presence. Moreover, these findings might still underestimate the visual realism of the avatars, as the experimental design required comparative assessment that might have aided their detection.

It is notable that to control for image detail in Experiment 1, both avatars and photographs were degraded using format-specific methods, comprising of mesh and texture simplification for avatars, and 2D resolution downsampling for photographs (see Method). It is possible that these distinct approaches introduce visual differences that might have affected categorisation of avatars and photos. This seems unlikely, however, as reduced image quality lowered the perceived realism for both avatars and faces, and increased the likelihood that both types of stimuli were categorised as avatars. This suggests that degrading quality blurred category boundaries irrespective of the method.

In Experiment 2, participants were then exclusively exposed to either avatars or photographs, commencing with the most realistic avatars from Experiment 1. This showed that this subset of avatars was perceived to be as realistic as genuine photographs, with 80.0% of participants failing to detect their presence spontaneously. Even when less realistic avatars were introduced subsequently, almost half of the participants (48.0%) still did not detect these stimuli. Moreover, some participants (16.0%) also mistook photographs for avatars, underscoring the challenge in distinguishing between these.

These findings highlight the effectiveness with which avatars can imitate real facial cues, even when designed with relative simplicity. For example, the avatars in this study were purposely kept minimalist, generated from original 3D scans with only fundamental positioning and lighting, without advanced rendering techniques, such as subsurface scattering, physically based shading, or path tracing. While this approach may limit the applicability of the findings to ‘hyper-realistic’ avatars, that might be even more difficult to distinguish from real faces, it provided a controlled level of digital realism. This also facilitated the direct comparison of observers’ perceptions of photographs and avatars based on judgements of attractiveness, dominance, and trustworthiness. In Experiment 3, these judgements converged between stimuli, reflecting that avatars and photographs were judged similarly. Additionally, only 36.7% of participants spontaneously noted the presence of avatars. In the fourth experiment we expanded this comparison by employing eye-tracking to investigate viewing patterns during realism and trait judgements. This revealed that the observation of avatars and photographs mirrors typical gaze patterns observed during face processing, with particular emphasis on the eyes, nose and mouth (e.g., Buchan et al., 2007; Henderson et al., 2001; Iskra & Gabrijelčič, 2016). This indicates that participants did not seek out *unusual* face cues in avatars than in photographs of real faces, and is consistent with the observation that spontaneous avatar detection was generally low. Indeed, only one consistent difference emerged in viewing behaviour, whereby avatars received about 5.0% more fixations to the eye-regions during all judgement types.

Taken together, these experiments demonstrate that the visual realism of these avatars developed for research is high. While these stimuli can be distinguished from photographs under forced-choice conditions or when observers are explicitly aware of avatar presence, the best avatars mimic *photo-realism*. These avatars achieve comparable realism ratings to photographs of real faces (Experiment 2), similar trait ratings as photographs (Experiment 3), and frequently go unnoticed when participants are unaware of the avatars’ inclusion in an experiment (Experiments 1 to 3).

These findings align with studies of other imitation technologies, such as hyper-realistic masks, deepfakes and AI-generated faces, which show that observers frequently mistake artificial stimuli for real faces (e.g., Miller et al., 2023; Sanders & Jenkins, 2018; Sanders et al., 2019). Although participants tend to rate avatar faces as less realistic than human faces in controlled comparisons, they rarely mention this difference spontaneously. This suggests that judgements of realism may often be processed implicitly, without rising to the level of conscious awareness – even when ratings reflect subtle perceptual differences, such as texture or lighting, that feel ‘off’ without being explicitly recognised as signs of digital generation.

Our study reinforces this interpretation: even when participants viewed only real photographs, some still expressed doubts about the authenticity of the faces in free-text responses. This could indicate that perceptions of artificiality can emerge independently of actual manipulations and may be influenced by baseline uncertainty in face perception. Sanders and Jenkins (2018) reported a comparable phenomenon, whereby participants frequently misclassified hyper-realistic masks as real and real faces as masks. Similarly, Will et al. (2021) manipulated levels of visual abstraction by embedding photographs of faces in other photographs. This ‘Medusa effect’ reduced the perceived realism of faces and mind attribution to their bearers, with these effects emerging spontaneously in eye-tracking patterns and behavioural responses, suggesting processing below the threshold of conscious awareness. Free-text responses in our study echo this: some participants expressed vague discomfort or noted atypical features (e.g., ‘eerie’ or ‘strange’) without identifying the faces as avatars, suggesting a sensed difference that was not necessarily mapped onto digital generation.

As digital avatars become increasingly integrated into and accepted as part of daily life (Gerlich, 2024), their perceived normalcy may further reduce the likelihood that individuals consciously register these person representations as artificial. Alternatively, individuals may be aware of avatars but not perceive these to be sufficiently out of the norm to comment on their presence. In this context, it is conceivable that the absence of spontaneous avatar-related comments in our study – even among those giving lower realism ratings – may reflect not a failure of detection, but a growing perceptual fluency with digital faces, which other studies are beginning to observe within younger generations (Tucciarelli et al., 2022).

The detection of some other face imitations, such as hyper-realistic masks, appears to rely disproportionately on the eye regions (Sanders & Jenkins, 2018). This also converges with the current study, in which avatars consistently attracted more fixations to the eye regions than real faces. One possible explanation for this difference might be that observers are particularly sensitive to the eye regions (e.g., Gilad et al., 2009) and therefore any differences between avatars and real faces might be easier to spot here than in other face regions. An alternative explanation is that visual differences between avatars and face photographs are particularly marked in the eye region, perhaps reflecting fine details, such as corneal reflections, to which observers may pay particular attention (Kätsyri et al., 2020). However, these effects appear to be subtle, accounting for only about 5% of fixations, and are consistent with the observation that unprompted avatar detection was low.

These subtle differences between the eye regions of avatars and face photographs might also explain some of the differences in attractiveness, trustworthiness and dominance ratings that were observed between experiments. These trait inferences were comparable for avatars and photographs in Experiment 3, in which observers only viewed one of these stimulus categories, whereas photographs received higher attractiveness and trustworthiness ratings than avatars in Experiment 4, in which avatars and photographs were intermixed. These differences between experiments might therefore reflect a combination of how the eye regions of faces contribute to these judgements and how subtle differences between avatars and photographs might become more apparent when a more direct comparison between these stimuli is possible. This finding converges with other studies, which show that first impressions of computer-generated faces and human faces are sometimes comparable, but computer-generated faces are also rated less favourably when realism differences are more perceptible (Miller et al., 2023). Other research has shown that trustworthiness judgements are influenced not only by visual features but also by perceived authenticity. Thus, both real and artificial faces are rated as less trustworthy when believed to be fake, regardless of their actual authenticity (Tucciarelli et al., 2022). This suggests that participants’ awareness of the stimuli's artificiality in Experiment 4 may have contributed to lower trait ratings for avatars, even though those avatars elicited similar impressions to real faces when viewed on their own (as in Experiment 3).

The eyes appear to be a focal point in attractiveness and trustworthiness judgements, surpassing other facial features such as the nose, mouth, eyebrows and jaw (Diego-Mas et al., 2020), whilst characteristics such as eye reflectance also serve to enhance attractiveness in computer-generated images of faces (Vaitonytė et al., 2021). When avatars and face photographs cannot be compared directly, as was the case with the between-subjects design of Experiment 3 in which observers were only shown one type of stimuli, then subtle differences between eye regions may go unnoticed and lead to comparable attractiveness and trustworthiness judgements. In a direct comparison, however, these differences may become more salient and undermine the trait evaluations of avatars.

In contrast to attractiveness and trustworthiness judgements, dominance ratings for avatars matched those for photographs in Experiment 4, suggesting that dominance perception relies on other facial features. This was evident in eye movements, which revealed more fixations on the nose region of photographs than avatars during dominance ratings. This aligns with previous research which suggests that the nose influences dominance perceptions more than other facial features (Diego-Mas et al., 2020). Dominance is also associated with other facial features, such as jaw shape (Diego-Mas et al., 2020; Oh et al., 2019) and higher facial width-to-height ratios (Carré & McCormick, 2008; Carré et al., 2009, 2010; Lefevre & Lewis, 2014; Merlhiot et al., 2021; Stirrat & Perrett, 2012). Therefore, dominance perceptions might rely more on facial structure than intricate details such as the eyes, which might explain why dominance judgements were comparable for photographs and avatars, even though avatars generated lower attractiveness and trustworthiness judgements in a direct comparison.

Finally, the current study also raises broader questions about factors that determine variation in realism ratings across different avatar identities. While the avatars in this study were generated from high-resolution 3D scans, not all were perceived to be equally realistic. It is possible that variation in scan fidelity – for example in features that are difficult to model accurately, such as hair, contributed to these differences. However, the eye-tracking data did not reveal increased attention to these areas, suggesting that other facial features or technical factors, such as subtle irregularities in skin texture, lighting, or the rendering of eyes, may also be influential. Understanding which features most affect perceived realism remains an open question. Moreover, since the current work focused on isolated, static faces, future studies might explore how realism perceptions change when avatars are embedded in more complex visual or social contexts, or when movement and body cues are introduced. Such factors influence the perception of real faces (see e.g., Brambilla et al., 2018; Freeman et al., 2013; Rice et al., 2013) and may also be critical for determining how digital faces are evaluated.

In summary, this study demonstrates that the visual realism of avatars created for scientific research can closely resemble that of face photographs, particularly in contexts in which realism is not explicitly evaluated. In contrast, differences between avatars and photographs become more apparent when participants are cognizant and able to make direct comparisons. Observers’ viewing patterns suggest that these differences might originate from the eye region of avatars, which consistently receive more fixations than those of photographs.

## Appendix A

**Table A.1.** Categorisation Comparisons – Face Type × Feature.

Comparison					Mean Difference	SE	*t*	*p*_Tukey_
Face Type	Feature		Face Type	Feature				
**Feature Within Photographs**								
Photograph	Eyes	–	Photograph	Nose	4.93	5.99	0.82	.999
		–	Photograph	Face	31.38	4.22	7.43	<.001
		–	Photograph	Mouth	34.81	4.39	7.93	<.001
		–	Photograph	Hair	40.85	3.48	11.73	<.001
		–	Photograph	Neck	41.86	3.48	12.02	<.001
	Nose	–	Photograph	Face	26.46	3.06	8.66	<.001
		–	Photograph	Mouth	29.88	3.50	8.54	<.001
		–	Photograph	Hair	35.92	3.11	11.56	<.001
		–	Photograph	Neck	36.93	2.98	12.39	<.001
	Face	–	Photograph	Mouth	3.42	1.35	2.53	.361
		–	Photograph	Hair	9.46	1.13	8.35	<.001
		–	Photograph	Neck	10.48	1.06	9.92	<.001
	Mouth	–	Photograph	Hair	6.04	1.56	3.87	.023
		–	Photograph	Neck	7.06	1.50	4.69	.003
	Hair	–	Photograph	Neck	1.01	0.38	2.69	.278
**Feature Within Avatars**								
Avatar	Eyes	–	Avatar	Nose	13.49	5.34	2.53	.364
		–	Avatar	Face	38.31	4.14	9.25	<.001
		–	Avatar	Mouth	41.73	4.19	9.97	<.001
		–	Avatar	Hair	47.50	3.22	14.73	<.001
		–	Avatar	Neck	48.05	3.27	14.69	<.001
	Nose	–	Avatar	Face	24.83	2.75	9.02	<.001
		–	Avatar	Mouth	28.25	2.91	9.69	<.001
		–	Avatar	Hair	34.02	2.59	13.11	<.001
		–	Avatar	Neck	34.56	2.51	13.76	<.001
	Face	–	Avatar	Mouth	3.42	1.22	2.81	.228
		–	Avatar	Hair	9.19	1.23	7.46	<.001
		–	Avatar	Neck	9.74	1.19	8.16	<.001
	Mouth	–	Avatar	Hair	5.77	1.41	4.08	.014
		–	Avatar	Neck	6.31	1.35	4.68	.003
	Hair	–	Avatar	Neck	0.55	0.22	2.46	.400
**Face Type Within Feature**								
Photograph	Eyes	–	Avatar	Eyes	−5.92	1.23	−4.83	.002
Photograph	Nose	–	Avatar	Nose	2.64	1.22	2.15	.592
Photograph	Face	–	Avatar	Face	1.01	0.71	1.43	.948
Photograph	Mouth	–	Avatar	Mouth	1.01	0.45	2.23	.542
Photograph	Hair	–	Avatar	Hair	0.73	0.46	1.61	.893
Photograph	Neck	–	Avatar	Neck	0.26	0.14	1.85	.779

*Note*. For all comparisons *df* = 29.

**Table A.2.** Attractiveness Rating Comparisons – Face Type × Feature.

Comparison					Mean Difference	*SE*	*t*	*p*_Tukey_
Face Type	Feature		Face Type	Feature				
**Feature Within Photographs**								
Photograph	Eyes	–	Photograph	Nose	8.29	5.44	1.52	.921
		–	Photograph	Face	33.39	4.52	7.39	<.001
		–	Photograph	Mouth	39.08	4.44	8.81	<.001
		–	Photograph	Hair	44.32	3.50	12.66	<.001
		–	Photograph	Neck	44.87	3.49	12.84	<.001
	Nose	–	Photograph	Face	25.10	2.02	12.42	<.001
		–	Photograph	Mouth	30.79	3.07	10.04	<.001
		–	Photograph	Hair	36.03	2.34	15.40	<.001
		–	Photograph	Neck	36.57	2.31	15.85	<.001
	Face	–	Photograph	Mouth	5.69	2.03	2.80	.230
		–	Photograph	Hair	10.94	1.23	8.91	<.001
		–	Photograph	Neck	11.48	1.26	9.14	<.001
	Mouth	–	Photograph	Hair	5.25	1.66	3.16	.115
		–	Photograph	Neck	5.79	1.66	3.49	.057
	Hair	–	Photograph	Neck	0.54	0.15	3.67	.038
**Feature Within Avatars**								
Avatar	Eyes	–	Avatar	Nose	17.70	5.19	3.41	.068
		–	Avatar	Face	38.55	4.53	8.52	<.001
		–	Avatar	Mouth	44.62	4.26	10.47	<.001
		–	Avatar	Hair	49.59	3.36	14.77	<.001
		–	Avatar	Neck	50.00	3.38	14.81	<.001
	Nose	–	Avatar	Face	20.85	2.20	9.49	<.001
		–	Avatar	Mouth	26.93	2.57	10.49	<.001
		–	Avatar	Hair	31.89	2.19	14.59	<.001
		–	Avatar	Neck	32.31	2.14	15.08	<.001
	Face	–	Avatar	Mouth	6.08	1.86	3.27	.093
		–	Avatar	Hair	11.04	1.47	7.52	<.001
		–	Avatar	Neck	11.46	1.46	7.86	<.001
	Mouth	–	Avatar	Hair	4.96	1.42	3.51	.055
		–	Avatar	Neck	5.38	1.42	3.80	.028
	Hair	–	Avatar	Neck	0.42	0.11	3.87	.023
**Face Type Within Feature**								
Photograph	Eyes	–	Avatar	Eyes	−5.10	1.26	−4.06	.014
Photograph	Nose	–	Avatar	Nose	4.30	1.32	3.26	.094
Photograph	Face	–	Avatar	Face	0.05	0.65	0.08	1.000
Photograph	Mouth	–	Avatar	Mouth	0.44	0.43	1.03	.996
Photograph	Hair	–	Avatar	Hair	0.16	0.17	0.92	.998
Photograph	Neck	–	Avatar	Neck	0.03	0.05	0.61	1.000

*Note*. For all comparisons *df* = 29.

**Table A.3.** Trustworthiness Rating Comparisons – Face Type × Feature.

Comparison					Mean Difference	*SE*	*t*	*p*_Tukey_
Face Type	Feature		Face Type	Feature				
**Feature Within Photographs**								
Photograph	Eyes	–	Photograph	Nose	10.73	5.53	1.94	.725
		–	Photograph	Face	35.56	4.11	8.66	<.001
		–	Photograph	Mouth	41.44	4.06	10.20	<.001
		–	Photograph	Hair	46.34	3.32	13.98	<.001
		–	Photograph	Neck	46.66	3.30	14.16	<.001
	Nose	–	Photograph	Face	24.83	2.44	10.19	<.001
		–	Photograph	Mouth	30.71	3.02	10.19	<.001
		–	Photograph	Hair	35.62	2.55	13.99	<.001
		–	Photograph	Neck	35.94	2.52	14.28	<.001
	Face	–	Photograph	Mouth	5.88	1.81	3.25	.095
		–	Photograph	Hair	10.78	1.13	9.53	<.001
		–	Photograph	Neck	11.10	1.15	9.66	<.001
	Mouth	–	Photograph	Hair	4.91	1.39	3.53	.052
		–	Photograph	Neck	5.23	1.43	3.65	.039
	Hair	–	Photograph	Neck	0.32	0.15	2.12	.616
**Feature Within Avatars**								
Avatar	Eyes	–	Avatar	Nose	18.13	4.92	3.69	.036
		–	Avatar	Face	40.69	3.79	10.73	<.001
		–	Avatar	Mouth	45.90	3.56	12.88	<.001
		–	Avatar	Hair	50.81	2.97	17.13	<.001
		–	Avatar	Neck	50.92	3.00	17.00	<.001
	Nose	–	Avatar	Face	22.56	2.14	10.54	<.001
		–	Avatar	Mouth	27.77	2.65	10.47	<.001
		–	Avatar	Hair	32.68	2.21	14.80	<.001
		–	Avatar	Neck	32.79	2.17	15.10	<.001
	Face	–	Avatar	Mouth	5.21	1.60	3.27	0.093
		–	Avatar	Hair	10.12	1.13	8.95	<.001
		–	Avatar	Neck	10.23	1.10	9.28	<.001
	Mouth	–	Avatar	Hair	4.91	1.20	4.08	.014
		–	Avatar	Neck	5.02	1.22	4.12	.013
	Hair	–	Avatar	Neck	0.11	0.12	0.94	.998
**Face Type Within Feature**								
Photograph	Eyes	–	Avatar	Eyes	−4.30	1.11	−3.87	.023
Photograph	Nose	–	Avatar	Nose	3.11	1.38	2.26	.527
Photograph	Face	–	Avatar	Face	0.83	0.77	1.09	.993
Photograph	Mouth	–	Avatar	Mouth	0.17	0.44	0.38	1.000
Photograph	Hair	–	Avatar	Hair	0.17	0.16	1.06	.994
Photograph	Neck	–	Avatar	Neck	−0.04	0.08	−0.51	1.000

*Note*. For all comparisons *df* = 29.

**Table A.4.** Dominance Rating Comparisons – Face Type × Feature.

Comparison					Mean Difference	*SE*	*t*	*p*_Tukey_
Face Type	Feature		Face Type	Feature				
**Feature Within Photographs**								
Photograph	Eyes	–	Photograph	Nose	7.57	5.43	1.40	.955
		–	Photograph	Face	35.07	4.25	8.26	<.001
		–	Photograph	Mouth	40.56	4.11	9.88	<.001
		–	Photograph	Hair	45.15	3.33	13.56	<.001
		–	Photograph	Neck	45.56	3.30	13.82	<.001
	Nose	–	Photograph	Face	27.49	2.39	11.50	<.001
		–	Photograph	Mouth	32.99	3.18	10.36	<.001
		–	Photograph	Hair	37.57	2.49	15.08	<.001
		–	Photograph	Neck	37.99	2.50	15.21	<.001
	Face	–	Photograph	Mouth	5.50	1.72	3.19	.109
		–	Photograph	Hair	10.08	1.18	8.51	<.001
		–	Photograph	Neck	10.49	1.19	8.79	<.001
	Mouth	–	Photograph	Hair	4.59	1.49	3.08	.135
		–	Photograph	Neck	5.00	1.52	3.30	.087
	Hair	–	Photograph	Neck	0.41	0.14	2.91	.188
**Feature Within Avatars**								
Avatar	Eyes	–	Avatar	Nose	17.41	4.75	3.67	.038
		–	Avatar	Face	40.74	3.67	11.12	<.001
		–	Avatar	Mouth	45.98	3.68	12.49	<.001
		–	Avatar	Hair	50.67	2.90	17.45	<.001
		–	Avatar	Neck	50.82	2.90	17.52	<.001
	Nose	–	Avatar	Face	23.33	1.97	11.83	<.001
		–	Avatar	Mouth	28.57	2.64	10.84	<.001
		–	Avatar	Hair	33.26	2.12	15.69	<.001
		–	Avatar	Neck	33.41	2.12	15.78	<.001
	Face	–	Avatar	Mouth	5.24	1.51	3.48	.058
		–	Avatar	Hair	9.93	1.00	9.98	<.001
		–	Avatar	Neck	10.08	0.99	10.23	<.001
	Mouth	–	Avatar	Hair	4.69	1.33	3.52	.053
		–	Avatar	Neck	4.84	1.35	3.59	.045
	Hair	–	Avatar	Neck	0.15	0.10	1.55	.914
**Face Type Within Feature**								
Photograph	Eyes	–	Avatar	Eyes	−5.30	1.07	−4.96	.001
Photograph	Nose	–	Avatar	Nose	4.54	1.08	4.20	.010
Photograph	Face	–	Avatar	Face	0.38	0.59	0.64	1.000
Photograph	Mouth	–	Avatar	Mouth	0.12	0.35	0.35	1.000
Photograph	Hair	–	Avatar	Hair	0.23	0.14	1.66	.873
Photograph	Neck	–	Avatar	Neck	−0.04	0.07	−0.48	1.000

*Note*. For all comparisons *df* = 29.

## References

[bibr1-03010066251379016] Althoff R. R. Cohen N. J. (1999). Eye-movement-based memory effect: A reprocessing effect in face perception. Journal of Experimental Psychology: Learning, Memory, and Cognition, 25(4), 997–1010. 10.1037/0278-7393.25.4.997 10439505

[bibr2-03010066251379016] Bindemann M. Fysh M. C. Trifonova I. V. Allen J. McCall C. Burton A. M. (2022). Face identification in the laboratory and in virtual worlds. Journal of Applied Research in Memory and Cognition, 11(1), 120–134. 10.1016/j.jarmac.2021.07.010

[bibr3-03010066251379016] Bindemann M. Scheepers C. Burton A. M. (2009). Viewpoint and center of gravity affect eye movements to human faces. Journal of Vision, 9(2), 1–16. 10.1167/9.2.7 19271917

[bibr4-03010066251379016] Brambilla M. Biella M. Freeman J. B. (2018). The influence of visual context on the evaluation of facial trustworthiness. Journal of Experimental Social Psychology, 78, 34–42. 10.1016/j.jesp.2018.04.011

[bibr5-03010066251379016] Buchan J. N. Paré M. Munhall K. G. (2007). Spatial statistics of gaze fixations during dynamic face processing. Social Neuroscience, 2(1), 1–13. 10.1080/17470910601043644 18633803

[bibr6-03010066251379016] Calder A. J. Young A. W. Perrett D. I. Etcoff N. L. Rowland D. (1996). Categorical perception of morphed facial expressions. Visual Cognition, 3(2), 81–118. 10.1080/713756735

[bibr7-03010066251379016] Carré J. M. McCormick C. M. (2008). In your face: Facial metrics predict aggressive behaviour in the laboratory and in varsity and professional hockey players. Proceedings of the Royal Society B: Biological Sciences, 275(1651), 2651–2656. 10.1098/rspb.2008.0873 PMC2570531 18713717

[bibr8-03010066251379016] Carré J. M. McCormick C. M. Mondloch C. J. (2009). Facial structure is a reliable cue of aggressive behavior. Psychological Science, 20(10), 1194–1198. 10.1111/j.1467-9280.2009.02423.x 19686297

[bibr9-03010066251379016] Carré J. M. Morrissey M. D. Mondloch C. J. McCormick C. M. (2010). Estimating aggression from emotionally neutral faces: Which facial cues are diagnostic? Perception, 39(3), 356–377. 10.1068/p6543 20465172

[bibr10-03010066251379016] Crookes K. Hayward W. G. (2012). Face inversion disproportionately disrupts sensitivity to vertical over horizontal changes in eye position. Journal of Experimental Psychology: Human Perception and Performance, 38(6), 1428–1437. 10.1037/a0027943 22506785

[bibr11-03010066251379016] Diego-Mas J. A. Fuentes-Hurtado F. Naranjo V. Alcañiz M. (2020). The influence of each facial feature on how we perceive and interpret human faces. I-Perception, 11(5). 10.1177/2041669520961123 PMC7533946 33062242

[bibr12-03010066251379016] Etcoff N. L. Magee J. J. (1992). Categorical perception of facial expressions. Cognition, 44(3), 227–240. 10.1016/0010-0277(92)90002-Y 1424493

[bibr13-03010066251379016] Freeman J. B. Ma Y. Han S. Ambady N. (2013). Influences of culture and visual context on real-time social categorization. Journal of Experimental Social Psychology, 49(2), 206–210. 10.1016/j.jesp.2012.10.015 23355750 PMC3551594

[bibr14-03010066251379016] Freire A. Lee K. Symons L. A. (2000). The face-inversion effect as a deficit in the encoding of configural information: Direct evidence. Perception, 29(2), 159–170. 10.1068/p3012 10820599

[bibr15-03010066251379016] Fysh M. C. Bindemann M. (2022). Molistic processing in facial image comparison. Applied Cognitive Psychology, 36(4), 830–841. 10.1002/acp.3975

[bibr16-03010066251379016] Fysh M. C. Trifonova I. V. Allen J. McCall C. Burton A. M. Bindemann M. (2022). Avatars with faces of real people: A construction method for scientific experiments in virtual reality. Behavior Research Methods, 54(3), 1461–1475. 10.3758/s13428-021-01676-5 34505276 PMC8428498

[bibr17-03010066251379016] Gerlich M. (2024). Societal perceptions and acceptance of virtual humans: Trust and ethics across different contexts. Social Sciences, 13(10), 516. 10.3390/socsci13100516

[bibr18-03010066251379016] Gilad S. Meng M. Sinha P. (2009). Role of ordinal contrast relationships in face encoding. Proceedings of the National Academy of Sciences, 106(13), 5353–5358. 10.1073/pnas.0812396106 PMC2664053 19276115

[bibr19-03010066251379016] Gobel M. S. Kim H. S. Richardson D. C. (2015). The dual function of social gaze. Cognition, 136, 359–364. 10.1016/j.cognition.2014.11.040 25540833

[bibr20-03010066251379016] Groh M. Epstein Z. Picard R. Firestone C. (2021). Human detection of deepfakes: A role for holistic face processing. Journal of Vision, 21(9), 2390. 10.1167/jov.21.9.2390

[bibr21-03010066251379016] Hancock J. T. Bailenson J. N. (2021). The social impact of deepfakes. Cyberpsychology, Behavior, and Social Networking, 24(3), 149–152. 10.1089/cyber.2021.29208.jth 33760669

[bibr22-03010066251379016] Henderson J. M. Falk R. Minur S. Dyer F. C. Mahadevan S. (2001). Gaze control for face learning and recognition by humans and machines. In Shipley T. F. Kellman P. J. (Eds.), From fragments to objects (Vol. 130, pp. 463–481). North-Holland. 10.1016/S0166-4115(01)80034-0

[bibr23-03010066251379016] Henderson J. M. Williams C. C. Falk R. J. (2005). Eye movements are functional during face learning. Memory & Cognition, 33(1), 98–106. 10.3758/BF03195300 15915796

[bibr24-03010066251379016] Iskra A. Gabrijelčič H. (2016). Eye-tracking analysis of face observing and face recognition. Journal of Graphic Engineering and Design, 7(1), 5–11. 10.24867/JGED-2016-1-005

[bibr25-03010066251379016] Jenkins R. White D. Van Montfort X. Mike Burton A. (2011). Variability in photos of the same face. Cognition, 121(3), 313–323. 10.1016/j.cognition.2011.08.001 21890124

[bibr26-03010066251379016] Kätsyri J. de Gelder B. de Borst A. W. (2020). Amygdala responds to direct gaze in real but not in computer-generated faces. NeuroImage, 204, 116216. 10.1016/j.neuroimage.2019.116216 31553928

[bibr27-03010066251379016] Klein J. T. Shepherd S. V. Platt M. L. (2009). Social attention and the brain. Current Biology, 19(20), R958–R962. 10.1016/j.cub.2009.08.010 PMC3387539 19889376

[bibr28-03010066251379016] Köbis N. C. Doležalová B. Soraperra I. (2021). Fooled twice: People cannot detect deepfakes but think they can. IScience, 24(11), 103364. 10.1016/j.isci.2021.103364 34820608 PMC8602050

[bibr29-03010066251379016] Lavan N. Mileva M. Burton A. M. Young A. W. McGettigan C. (2021). Trait evaluations of faces and voices: Comparing within- and between-person variability. Journal of Experimental Psychology: General, 150(9), 1854–1869. 10.1037/xge0001019 33734774 PMC7612101

[bibr30-03010066251379016] Leder H. Bruce V. (2000). When inverted faces are recognized: The role of configural information in face recognition. The Quarterly Journal of Experimental Psychology Section A, 53(2), 513–536. 10.1080/713755889 10881616

[bibr31-03010066251379016] Lefevre C. E. Lewis G. J. (2014). Perceiving aggression from facial structure: Further evidence for a positive association with facial width–to–height ratio and masculinity, but not for moderation by self–reported dominance. European Journal of Personality, 28(6), 530–537. 10.1002/per.1942

[bibr32-03010066251379016] Leopold D. A. Rhodes G. (2010). A comparative view of face perception. Journal of Comparative Psychology, 124(3), 233–251. 10.1037/a0019460 20695655 PMC2998394

[bibr33-03010066251379016] McKone E. Martini P. Nakayama K. (2001). Categorical perception of face identity in noise isolates configural processing. Journal of Experimental Psychology: Human Perception and Performance, 27(3), 573–599. 10.1037/0096-1523.27.3.573 11424647

[bibr34-03010066251379016] Merlhiot G. Mondillon L. Méot A. Dutheil F. Mermillod M. (2021). Facial width-to-height ratio underlies perceived dominance on facial emotional expressions. Personality and Individual Differences, 172, 110583. 10.1016/j.paid.2020.110583

[bibr35-03010066251379016] Mileva M. Young A. W. Kramer R. S. S. Burton A. M. (2019). Understanding facial impressions between and within identities. Cognition, 190, 184–198. 10.1016/j.cognition.2019.04.027 31102977

[bibr36-03010066251379016] Miller E. J. Foo Y. Z. Mewton P. Dawel A. (2023). How do people respond to computer-generated versus human faces? A systematic review and meta-analyses. Computers in Human Behavior Reports, 10, 100283. 10.1016/j.chbr.2023.100283

[bibr37-03010066251379016] Nielsen M. K. Slade L. Levy J. P. Holmes A. (2015). Inclined to see it your way: Do altercentric intrusion effects in visual perspective taking reflect an intrinsically social process? Quarterly Journal of Experimental Psychology, 68(10), 1931–1951. 10.1080/17470218.2015.1023206 25849956

[bibr38-03010066251379016] O’Grady C. Scott-Phillips T. Lavelle S. Smith K. (2020). Perspective-taking is spontaneous but not automatic. Quarterly Journal of Experimental Psychology, 73(10), 1605–1628. 10.1177/1747021820942479 PMC7551223 32718242

[bibr39-03010066251379016] Oh D. Dotsch R. Todorov A. (2019). Contributions of shape and reflectance information to social judgments from faces. Vision Research, 165, 131–142. 10.1016/j.visres.2019.10.010 31734634

[bibr40-03010066251379016] Oosterhof N. N. Todorov A. (2008). The functional basis of face evaluation. Proceedings of the National Academy of Sciences, 105(32), 11087–11092. 10.1073/pnas.0805664105 PMC2516255 18685089

[bibr41-03010066251379016] Pan X. Hamilton A. F. C. (2018). Why and how to use virtual reality to study human social interaction: The challenges of exploring a new research landscape. British Journal of Psychology, 109(3), 395–417. 10.1111/bjop.12290 29504117 PMC6055846

[bibr42-03010066251379016] Rayner K. Pollatsek A. (1994). The psychology of reading (1st ed.). Routledge. 10.4324/9780203357798

[bibr43-03010066251379016] Rice A. Phillips P. J. Natu V. An X. O’Toole A. J. (2013). Unaware person recognition from the body when face identification fails. Psychological Science, 24(11), 2235–2243. 10.1177/0956797613492986 24068115

[bibr44-03010066251379016] Robertson D. J. Sanders J. G. Towler A. Kramer R. S. S. Spowage J. Byrne A. Burton A. M. Jenkins R. (2020). Hyper-realistic face masks in a live passport-checking task. Perception, 49(3), 298–309. 10.1177/0301006620904614 32013720 PMC7583446

[bibr45-03010066251379016] Sanders J. G. Jenkins R. (2018). Individual differences in hyper-realistic mask detection. Cognitive Research: Principles and Implications, 3(1), 24. 10.1186/s41235-018-0118-3 30009254 PMC6019421

[bibr46-03010066251379016] Sanders J. G. Jenkins R. (2021). Realistic masks in the real world. In Bindemann M. (Ed.), Forensic face matching (pp. 216–236). Oxford University Press. 10.1093/oso/9780198837749.003.0010

[bibr47-03010066251379016] Sanders J. G. Ueda Y. Minemoto K. Noyes E. Yoshikawa S. Jenkins R. (2017). Hyper-realistic face masks: A new challenge in person identification. Cognitive Research: Principles and Implications, 2(1), 43. 10.1186/s41235-017-0079-y 29104914 PMC5655619

[bibr48-03010066251379016] Sanders J. G. Ueda Y. Yoshikawa S. Jenkins R. (2019). More human than human: A Turing test for photographed faces. Cognitive Research: Principles and Implications, 4(1), 1–10. 10.1186/s41235-019-0197-9 31748844 PMC6868074

[bibr49-03010066251379016] Schilbach L. (2015). Eye to eye, face to face and brain to brain: Novel approaches to study the behavioral dynamics and neural mechanisms of social interactions. Current Opinion in Behavioral Sciences, 3, 130–135. 10.1016/j.cobeha.2015.03.006

[bibr50-03010066251379016] Stirrat M. Perrett D. I. (2012). Face structure predicts cooperation: Men with wider faces are more generous to their in-group when out-group competition is salient. Psychological Science, 23(7), 718–722. 10.1177/0956797611435133 22623509

[bibr51-03010066251379016] Sutherland C. A. M. Oldmeadow J. A. Santos I. M. Towler J. Michael Burt D. Young A. W. (2013). Social inferences from faces: Ambient images generate a three-dimensional model. Cognition, 127(1), 105–118. 10.1016/j.cognition.2012.12.001 23376296

[bibr52-03010066251379016] Todorov A. Pakrashi M. Oosterhof N. N . (2009). Evaluating faces on trustworthiness after minimal time exposure. Social Cognition, 27(6), 813–833. 10.1521/soco.2009.27.6.813

[bibr53-03010066251379016] Todorov A. Porter J. M. (2014). Misleading first impressions. Psychological Science, 25(7), 1404–1417. 10.1177/0956797614532474 24866921

[bibr54-03010066251379016] Tucciarelli R. Vehar N. Chandaria S. Tsakiris M. (2022). On the realness of people who do not exist: The social processing of artificial faces. IScience, 25(12), 105441. 10.1016/j.isci.2022.105441 36590465 PMC9801245

[bibr55-03010066251379016] Turing A. M. (1950). Computing intelligence and machinery. Mind A Quarterly Review of Psychology and Philosophy, 59(236), 433–460. 10.1093/mind/LIX.236.433

[bibr56-03010066251379016] Vaitonytė J. Blomsma P. A. Alimardani M. Louwerse M. M. (2021). Realism of the face lies in skin and eyes: Evidence from virtual and human agents. Computers in Human Behavior Reports, 3, 100065. 10.1016/j.chbr.2021.100065

[bibr57-03010066251379016] Will P. Merritt E. Jenkins R. Kingstone A. (2021). The Medusa effect reveals levels of mind perception in pictures. Proceedings of the National Academy of Sciences, 118(32). 10.1073/PNAS.2106640118 PMC8364175 34353914

[bibr58-03010066251379016] Willis J. Todorov A. (2006). First impressions: Making up your mind after a 100-ms exposure to a face. Psychological Science, 17(7), 592–598. 10.1111/j.1467-9280.2006.01750.x 16866745

[bibr59-03010066251379016] Young A. W. (2018). Faces, people and the brain: The 45th sir frederic bartlett lecture. Quarterly Journal of Experimental Psychology, 71(3), 569–594. 10.1177/1747021817740275 29461174

